# The Multipartite Mitochondrial Genome of Marama (*Tylosema esculentum*)

**DOI:** 10.3389/fpls.2021.787443

**Published:** 2021-12-08

**Authors:** Jin Li, Christopher Cullis

**Affiliations:** Department of Biology, Case Western Reserve University, Cleveland, OH, United States

**Keywords:** *Tylosema esculentum*, marama, Fabaceae, mitochondrial genome sequence, hybrid genome assembly, genome sequencing, homologous recombination, multipartite genomic conformations

## Abstract

*Tylosema esculentum* (marama bean), a wild legume from tropical Africa, has long been considered as a potential crop for local farmers due to its rich nutritional value. Genomics research of marama is indispensable for the domestication and varietal improvement of the bean. The chloroplast genome of marama has been sequenced and assembled previously using a hybrid approach based on both Illumina and PacBio data. In this study, a similar method was used to assemble the mitochondrial genome of marama. The mitochondrial genome of the experimental individual has been confirmed to have two large circles OK638188 and OK638189, which do not recombine according to the data. However, they may be able to restructure into five smaller circles through recombination on the 4 pairs of long repeats (>1 kb). The total length of marama mitogenome is 399,572 bp. A 9,798 bp DNA fragment has been found that is homologous to the chloroplast genome of marama, accounting for 2.5% of the mitogenome. In the Fabaceae family, the mitogenome of *Millettia pinnata* is highly similar to marama, including for both the genes present and the total size. Some genes including *cox2, rpl10, rps1*, and *sdh4* have been lost during the evolution of angiosperms and are absent in the mitogenomes of some legumes. However, these remain intact and functional in marama. Another set of genes, *rpl2, rps2, rps7, rps11*, *rps13*, and *rps19* are either absent, or present as pseudogenes, in the mitogenome of marama.

## Introduction

**FIGURE 1:**
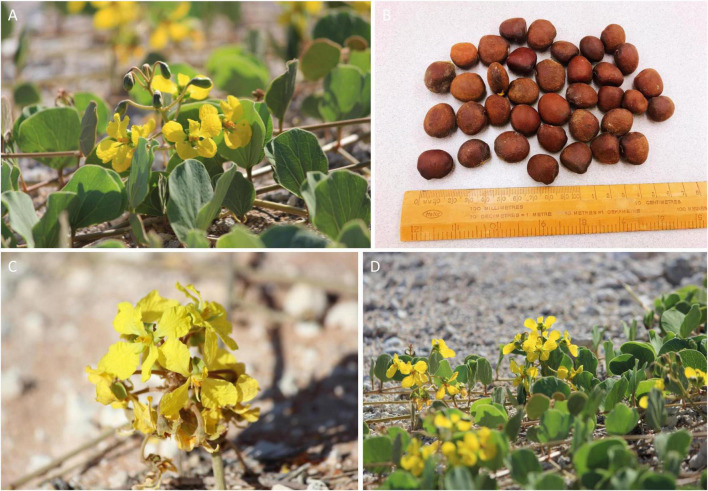
Morphology of marama (*Tylosema esculentum*) plants from Namibia. **(A)** Double-lobed leaves, soft and red-brown when young, become leathery and gray-green over time (National Research Council [NRC], 2006). **(B)** Brownish-black seeds, edible after roasting and rich in protein, lipids, and micronutrients (Jackson et al., 2010). **(C)** Yellow-orange flowers, bloom every midsummer from the 3rd or 4th year after planting (Bower et al., 1988). **(D)** Prostrate vines, can grow up to six meters or longer for mature plants (Vietmeyer, 1986).

*Tylosema esculentum*, also known as gemsbok bean, tamani berry, and marama bean, is a long-lived perennial legume native to the Kalahari Desert and adjacent arid and semi-arid regions in Botswana, Namibia, and South Africa (Bower et al., 1988; Figure 1). Marama naturally grows in environments of long-term drought, low rainfall (100–900 mm), and extreme high temperatures (daily maximum of 37°C in the growing season) (van der Maesen, 2006). Marama is often referred to as “the green gold of Africa,” and has long been considered as a potential crop due to its highly nutritious seeds and tubers.

The protein content of marama seeds reaches 30–39% dry matter (dm) (Belitz et al., 2004; Dakora, 2013) and the lipid content ranges from 35 to 48% dm (Amarteifio and Moholo, 1998), both of which are comparable to commercial crops like soybean and peanut (Belitz et al., 2004). Marama also provides many micronutrients including calcium, iron, zinc, phosphate, magnesium and B vitamins (folate), as well as various phytonutrients, such as phenolic compounds (tannins), trypsin inhibitors, phytates and oligosaccharides, which can help prevent cardiovascular diseases, diabetes, and certain cancers (Jackson et al., 2010).

Despite its social-economic value, as an orphan crop, marama is still underdeveloped in terms of research and breeding. Domesticating marama and increasing its seed yield could increase the food security in arid zones where traditional crops are not suitable for cultivation, and diversify the livelihoods of people living there.

Marama is a basal legume that does not form nodules, which may be beneficial for drought avoidance and make the plant better adapted to arid environments (Cullis et al., 2018). The genome size of marama was estimated at about 1 gigabase through Feulgen staining (Takundwa et al., 2012). The number of chromosomes is 42 and it is speculated that marama is a hexaploid (7 chromosomes for haploid) (Takundwa et al., 2012). The complete *T. esculentum* chloroplast genome was assembled and found to be 161,537 bp in length and contains a specific inversion of 7,479 bp, which includes the six genes *rbcL*, *accD*, *psaI*, *ycf4*, *cemA*, and *petA*, and has not been found in other legumes (Kim and Cullis, 2017). A large amount of genetic diversity was found in Namibian marama germplasm within populations, but no significant differences between populations, through assessment using SSR markers, and the level of genetic diversity was thought to be related to the plant growth environment and stress response (Nepolo, 2010).

As the powerhouse of cells, mitochondria are where oxidative phosphorylation takes place and the cellular energy source adenosine triphosphate (ATP) is generated (Siekevitz, 1957). Mitochondria play an important role in the growth and development of organisms by regulating respiration, metabolism, programmed cell death (PCD) (Galluzzi et al., 2012) and intracellular signaling (Epstein et al., 2001). Mitochondrial defects are associated with cytoplasmic male sterility (CMS) in plants, preventing them from producing functional pollen. This can be utilized by breeders to ensure crossing of self-pollinating plants and obtain hybrid seeds (Kempken and Pring, 1999). Mitochondrial function is also believed to be related to the adaptability of organisms to the environment and their response to abiotic stress. For example, during the development of mung bean (*Vigna radiata*) cotyledons, *in vivo* freeze-thaw treatment converted the mitochondrial rosette genome structure to a longer linear DNA shown by electron microscopy (Cheng et al., 2016). Knowing whether different mtDNA structures coexist in marama individuals and the transition between them may provide information to understand better why marama can perform well in harsh environments. In addition, since plant mitochondrial genes are highly conserved with a low nucleotide mutation rate, they can be used in the study of plant evolution (Palmer and Herbon, 1988).

Self-incompatibility (SI) is a common genetically determined mechanism in flowering plants that promotes outcrossing and prevents inbreeding to increase genetic diversity. Marama is self-incompatible. This is one major obstacle to its cultivation and breeding, since SI not only reduces the yield of seeds and/or fruits for crops (Miller and Gross, 2011), but also makes it extremely difficult to combine desirable traits of two incompatible parents through simple cross-pollination (Claessen et al., 2019). The interaction between NaSIPP, a mitochondrial protein with phosphate transporter activity, exclusively transcribed in mature pollen and stigma protein NaStEP has been found essential to SI in *Nicotiana* spp., and this may destabilize mitochondria and stop pollen tube growth (García-Valencia et al., 2017). Since most self-incompatible Fabaceae plants are believed to apply a similar gametophytic SI system, the sequencing of marama mtDNA is indispensable for studying marama self-incompatibility (Aguiar et al., 2015).

Plant mitochondrial genomes are very complex and diverse in size, sequence arrangement, repeat content and structure (Kubo and Mikami, 2007; Mower et al., 2012). Unlike animal mitochondrial genomes, which are usually only 16–20 kb, the mitochondrial genome of higher plants can vary from 200 to 2,000 kb in size (Fauron et al., 1995). Some newly sequenced plant mitogenomes exceed this range, even reaching 11.3 Mb in the flowering plant *Silene conica* (Sloan et al., 2012). Although many plant mitochondrial genomes can be combined into one ring, in fact, their physical structure may be very complex and composed of multiple circular, linear, and branched molecules suggested by electron microscopy (Backert et al., 1997). There is a large number of repeats in plant mitochondrial genomes, including long repeats (>1 kb), short repeats (<100 nt), and tandem repeats. Repeat-mediated recombination is believed to be the main cause of changes in the structure of mtDNA and plays an important role in mitochondrial replication and repair (Maréchal and Brisson, 2010). Although the size of plant mitogenomes is often large, their gene pool (24 core genes with 17 variable genes) is usually small, since many genes have been either lost or transferred to nucleus during angiosperm evolution, but the coding sequences of the remaining genes are highly conserved (Adams et al., 2002).

The existence of many repetitive sequences and multipartite subgenomes makes the assembly of plant mitochondrial genome difficult. The hybrid assembly approach using PacBio long reads to fill the gaps together with high coverage Illumina short reads to correct sequencing errors has, however, proven to be effective in accomplishing this task (Kozik et al., 2019). In this study, a similar method has been used to unveil the mitochondrial genome of marama.

## Materials and Methods

### Plant Materials Sources and Genome Sequencing

The marama samples were collected from plants growing at the University of Pretoria Farm in South Africa, and from various locations in Namibia. Total DNA was extracted from fresh young leaves using a Qiagen Plant miniprep kit following the manufacturer’s protocol. The purity of DNA samples was assessed using a NanoDrop spectrophotometer to measure the ratio of 260/280 and 260/230 and was confirmed by electrophoresing 20 μL of the sample on a 1.0% agarose TBE gel at 100 V running for 50 min, using GelRed^®^ for DNA staining. The samples were then sent to the Génome Québec Innovation Centre, CWRU Genomics Core and Novogene Corporation for the Illumina sequencing and the PacBio RSII SMRT/Québec platform was used to generate the long-read sequencing data. 179,470,509 reads covering 26.9 Gb were generated from the Illumina HiSeq 2000 platform for an individual collected in Namibia and the data was used for the first round assembly due to the high coverage. 21,373,859 reads, 37,816,777 reads and 46,425,865 reads were obtained from the same Illumina platform for one individual growing at the University of Pretoria Farm and two other plants grown from seeds collected in Namibia. The sample from the University of Pretoria Farm was also sequenced in five PacBio SMRT cells, producing 1.78 Gb of sequence with an average coverage of 15×–20× for the mitogenomes. All raw Illumina and PacBio reads were submitted to the NCBI SRA database^1^ under the accession number PRJNA779273.

### Mitochondrial Genome Assembly

The paired end Illumina reads were assembled *de novo* by ABySS, which were further elongated using DBG2OLC to get the preliminary genome assembly. The mitochondrial contigs were retrieved based on the alignment and BLAST with known plant’s, especially Fabaceae, mitogenomes. Ten mitochondrial genomes from 7 legumes and 3 other model plants including *Arabidopsis thaliana, Glycine max, Lotus japonicas, Medicago truncatula, Millettia pinnata, Vicia faba, Vigna angularis, Vigna radiate*, and *Zea mays (2)* were retrieved from NCBI Organelle Genome Resources. The Illumina reads of the studied individual were aligned to the 10 mitogenomes by Bowtie 2 using the i-Plant Discovery Environment (Cyverse).

The mitochondrial genome of *Millettia pinnata* (NC_016742.1) showed the highest alignment rate and was used as the reference to recover the marama mitochondrial genome. The coding sequences of *Millettia pinnata* mitochondrial genes including *atp1, atp4, atp6, atp8, atp9, ccmB, ccmC, ccmFc, ccmFn, cob, cox1, cox2, cox3, matR, mttB, nad1, nad2, nad3, nad4, nad4L, nad5, nad6, nad7, nad9, rpl2, rpl5, rpl16, rps1, rps2, rps3, rps4, rps7, rps10, rps11, rps12, rps14, rps19, sdh3*, and *sdh4* were obtained from NCBI GenBank and used to find corresponding homologs in the marama genome assembly using BLAST. The acquired homologous sequences were then extended and assembled manually based on both the Illumina and PacBio sequencing data. A 500 bp sequence at both ends of each contig was BLASTed against the PacBio database on Geneious 9, and all obtained results were BLASTed back to the genome assembly to show all the possible connections. The ID and number of PacBio reads going across each contig junction were recorded, manually counted and saved in a document. Two long sequences were eventually generated from these steps.

The Illumina reads of the sample from the University of Pretoria Farm were aligned to the two assembled sequences using Bowtie 2. In total, 16 primary scaffolds were obtained and then they were used for the second round of assembly. The process was repeated until the complete mitochondrial genome of marama has been recovered. The results were verified through alignment using the Illumina reads of other marama individuals against the assembled sequences by Bowtie 2 and the alignments were visualized in IGV.

The PacBio reads were assembled directly using Canu v2.2 (correctedErrorRate = 0.15, genomeSize = 20 m) to further verify the mitogenomic structures obtained.

### Gene Annotation

The protein coding and rRNA genes were annotated using MITOFY^2^ and AGORA^3^. The two programs gave similar annotation results including gene sets, positions and matching rates, which were summarized and recorded in one spreadsheet. MITOFY BLASTN was used to annotate tRNA genes in the assembled genome. The sequence of the two circles comprising the marama mitochondrial genome and corresponding annotations were uploaded to GenBank under the accession numbers OK638188 and OK638189. The circular visualization of the gene annotation was generated using OGDRAW^4^.

## Results and Discussion

The Bowtie2 alignments resulted in 0.48% of the marama Illumina reads mapping to the mitochondrial genome of *Millettia pinnata*, 0.43% to *Lotus japonicus*, 0.31% to *Glycine max* and 0.30% to *Vigna radiate*. Therefore, the homologs of *Millettia pinnata* mitochondrial genes in the marama genome assembly were used as the starting point for the assembly.

**FIGURE 2:**
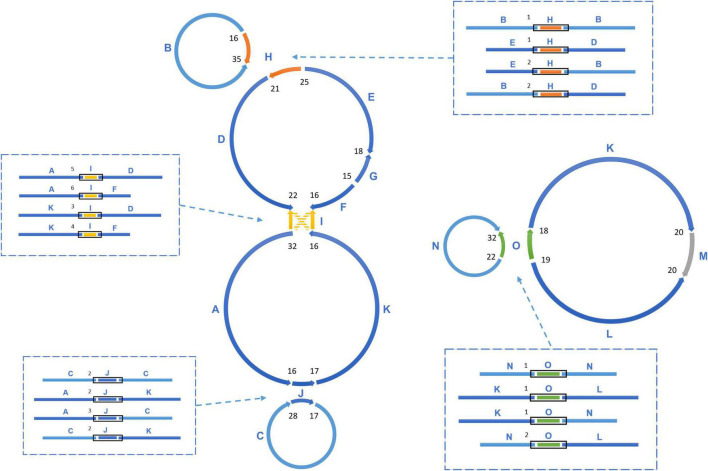
The assembly graph of multipartite *T. esculentum* mitochondrial genome. The marama mitochondrial genome is composed of 5 circular molecules, which were built based on 16 primary long scaffolds. 4 of them shown here including H, I, J, O are long repeats, which are present in two copies in the marama mitogenome. The scaffolds are assembled together by the PacBio long reads across the gaps. Connections between two adjacent scaffolds were quantified by PacBio reads and the counts are shown as numbers in black. Recombination on repeats causes the existence of different connections between the same scaffolds, which are shown (within black boxes) in the dashed boxes and also quantified by PacBio reads. The numbers next to the black boxes represent their counts. According to the connections shown in the dashed boxes, the 16 scaffolds can also be assembled into two long rings, as shown in Figure 3. The PacBio long reads data confirmed that these two structures co-exist in the experimental individual and they are close to the same frequency.

**FIGURE 3:**
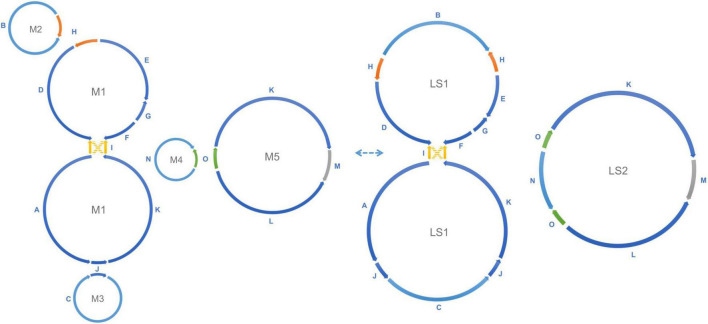
Recombination between repeats forms alternative mitochondrial genomic conformations. Recombination across the direct repeats H (orange), J (blue), and O (green) merges the five small circular molecules into two large rings. M2 and the upper ring of M1 are merged into the upper ring of LS1, the lower ring of M1 and M3 form the lower ring of LS1, and the M4 and M5 are merged into LS2, as shown in the figure. This transformation is reversible. Recombination between a single pair of inverted repeats I (yellow) changes the connection between the upper M1/LS1 ring and the lower M1/LS1 ring (AID, KIF to AIF, KID). The connection shown by the yellow solid line and the connection shown by the yellow dashed line are interchangeable through recombination. All structures mentioned above are experimentally verified in the data.

The sequencing data was initially assembled into 16 primary scaffolds, varying in size from 2,351 to 56,817 bp with a median of 27,406 bp (Table 1). According to the PacBio long reads at the junctions, these scaffolds joined to form five possible circular molecules, the sizes of which are 169,330, 44,455, 39,474, 32,520, and 113,793 bp (Figure 2 and Table 2). The Illumina short reads were remapped to the final assembly using Bowtie 2 and the proportion of mitochondrial reads in the whole genome sequence data varied from 2 to 3% between individuals. Four large repeats of 5,212, 2,351, 3,908, and 4,926 bp were found in these molecules (H, I, J, and O on Figure 2), accounting for 8.2% of the genome (Table 1 and Figure 2). The depths of coverage of these four regions are double compared to the single copy sequences (Supplementary Figures 1–4). Four long fragments with lengths of 9,798 bp (M on Figure 2), 859, 342, and 273 bp in marama mtDNA were identified as homologous to regions of the marama chloroplast genome with similarity ranging from 74 to 98%, covering 7.2% of the chloroplast genome and 2.8% of the mitochondrial genome. These fragments are all on one circular molecule (M5 on Figure 3) and contain some chloroplast genes including *psaA*, *psaB* and part of *psbC* (Supplementary Figure 5). When these chloroplast insertions occurred in the evolution and their role in the mitogenome remains unknown.

**TABLE 1:** Lengths of primary scaffolds used for the assembly of *T. esculentum* mitochondrial genome.

Unit number	Length (bp)	Unit number	Length (bp)
A	39,443	N	27,635
J	3,908	O	4,926
K	39,470	K	56,817
C	35,384	L	42,335
B	39,313	M	9,760
D	34,472		
H	5,212		
E	27,177		
G	5,443		
F	8,311		
I	2,351		

**TABLE 2:** Summary of *T. esculentum* mitochondrial subgenome features.

Molecule	A (%)	C (%)	G (%)	T (%)	G∼C (%)	Length (bp)
M1	27.88	22.14	22.26	27.72	44.40	169,330
M2	27.67	21.68	23.18	27.47	44.86	44,455
M3	27.87	22.23	23.06	26.84	45.29	39,474
M4	27.76	21.65	22.83	27.76	44.48	32,520
M5	27.29	22.43	22.57	27.71	45.00	113,793
LS1	27.81	22.34	22.28	27.58	44.62	253,259
LS2	27.39	22.52	22.36	27.72	44.88	146,313

Homologous recombination between long repeats can reorganize the plant mitochondrial genome structure causing the formation of different subgenomic molecules. Two large circular molecules have been confirmed existing in the marama mitochondrial genome, and they may be able to reversibly transform into the five basic molecules through such recombination (Figure 3). No connection was found between the two large molecules in the mitochondrial genome of the experimental marama individual. 12 long contigs were obtained from the direct assembly of PacBio reads using Canu v2.2 and the contigs were aligned to the structure units of LS1 and LS2 using BLAST to verify the connections as shown in Supplementary Figures 6–17. However, whether they can further combine to form one large circular molecule like the mtDNA of other legumes including *Millettia pinnata*, *Vigna radiate*, and *Glycine max* (Alverson et al., 2011; Kazakoff et al., 2012; Chang et al., 2013) in other marama individuals still needs to be determined. In addition, from the PacBio data, the ratio of five basic rings to the two large molecules was found to be approximately 1:1 (Figure 2), but the sample size is very small and more PacBio data or qPCR amplifications at the junctions are required to verify the ratios.

**FIGURE 4:**
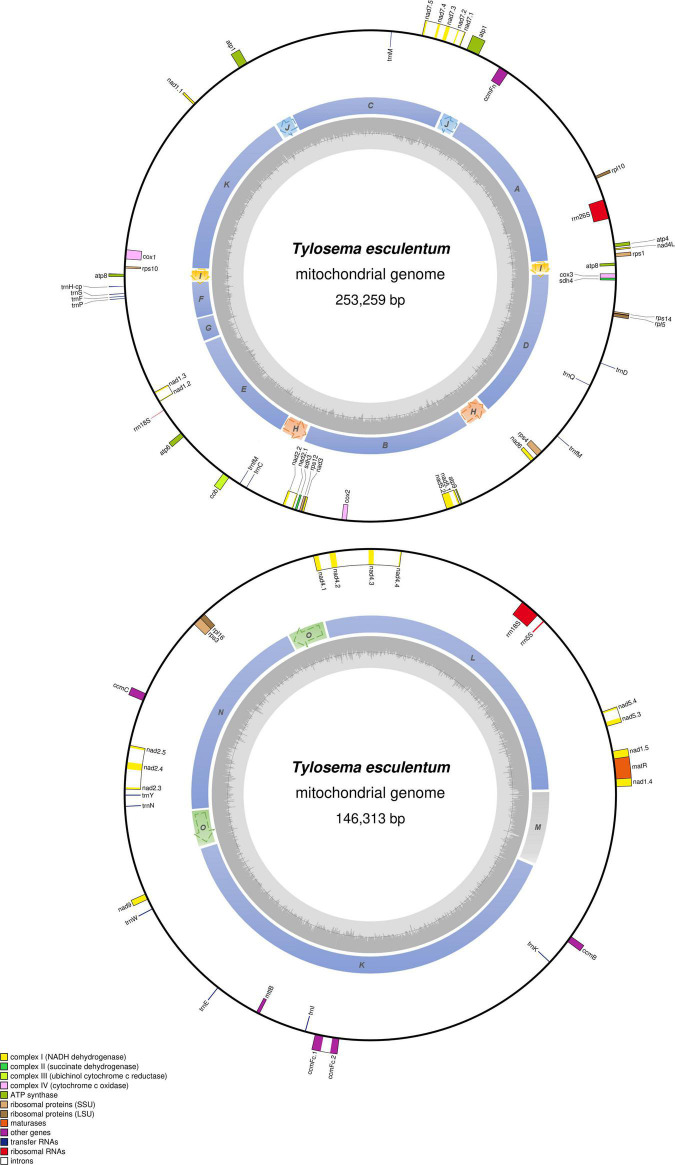
Circular gene map of the two large molecules, LS1 **(top)** and LS2 **(bottom)** in the mitochondrial genome of *T. esculentum.* Genes outside the circle are transcribed clockwise, while genes inside are transcribed counterclockwise. The sequential order of exons is displayed by the decimal after the gene name abbreviation. Genes belonging to different functional groups are color-coded as shown at bottom left. Colors in the inner circle indicate the position of the structural units and repeats listed in Figure 3. The dashed arrows represent the direction of the repeats. GC content is represented by the gray shade in the inner circle.

The total length of the marama mitochondrial genome is 399,572 bp, which is slightly shorter than the mitochondrial genomes of some other Fabaceae, 402,558 bp for *Glycine max*, 401,262 bp for *Vigna radiate* and 425,718 bp for *Millettia pinnata* (Alverson et al., 2011; Kazakoff et al., 2012; Chang et al., 2013; Negruk, 2013), but larger than *Arabidopsis thaliana* (366,924 bp) (Unseld et al., 1997). The GC content is 44.71%, close to 44.8% of *Arabidopsis thaliana* (Unseld et al., 1997), 45.4% of *Millettia pinnata* (Kazakoff et al., 2012), and 45.1% of *Vigna radiata* (Alverson et al., 2011). 35 protein coding genes, 3 rRNA genes, and 15 tRNA genes were identified from the assembled sequences using MITOFY and AGORA organelle genome annotation platforms (Table 3) and the positions are shown in Figure 4. Four protein coding genes *rpl2*, *rps2*, *rps11*, and *rps13* are completely missing from the mitochondrial genome of marama (Table 4). Two protein coding genes *rps7* and *rps19* are present as pseudogenes since each contains one internal stop codon. Genes *atp1*, *atp8* and one open reading frame (ORF) of *nad6* have two copies since they are on long repeats H, J, and I separately. The two copies of *atp1* have ORFs of different lengths since both the stop codons are outside the repeated sequence (Supplementary Figure 18). Whether marama mtDNA recombination and structural variation have altered the expression of these genes remains to be determined. Genes including *nad1*, *nad2*, *nad4*, *nad5*, *nad7*, and *ccmFc* contain introns as shown in Figure 4. The tRNA gene *trnfM-CAT* has two copies in the marama mitochondrial genome, while it has four copies in the mitogenomes of soybean (Chang et al., 2013). Some genes have been lost during angiosperm evolution (Table 4). For example, *rpl10* is absent from the mitochondrial genomes of *Millettia pinnata* and *Vigna radiata*, however, it is present and functional in marama (Alverson et al., 2011; Kazakoff et al., 2012). Genes including *cox2* and *rps1* are missing from the mtDNA of *Vigna radiata* and *Lotus japonicus*, but remain intact in marama, *Millettia pinnata*, and *Glycine max* mtDNA (Kazakoff et al., 2012; Chang et al., 2013). *sdh4* exists as a pseudogene in *Millettia pinnata* and soybean, but it was found to be functional in the mitogenome of marama (Kazakoff et al., 2012; Chang et al., 2013). A phylogenetic tree was drawn based on 29 chloroplast protein coding genes to study the relationship between marama and other related plants (Kim and Cullis, 2017). A similar phylogenetic study can be carried out in the future according to the mitochondrial genes mentioned above to further verify the taxonomic relationship between marama and other Fabaceae, which will provide an in-depth understanding of how marama has evolved.

**TABLE 3:** Annotated genes in the mitochondrial genomes of *T. esculentum*.

Category	Names of genes
Complex I (NADH dehydrogenase)	*nad1-7*, *nad4L*, and *nad9*
Complex II (succinate dehydrogenase)	*sdh3*, *sdh4*
Complex III (ubiquinol cytochrome-c reductase)	*cob*
Complex IV (cytochrome-c oxidase)	*cox1-3*
Complex V (ATP synthase)	*atp1*, *atp4*, *atp6*, *atp8*, *atp9*
Cytochrome c biogenesis	*ccmB*, *ccmC*, *ccmFc*, *ccmFn*
Large subunit ribosomal proteins	*rpl5*, *rpl10*, *rpl16*
Small subunit ribosomal proteins	*rps1*, *rps3*, *rps4*, *rps10*, *rps12*, *rps14*
Maturases	*matR*
Transport membrane protein	*mttB*
Ribosomal RNAs	*rrn5, rrn26, rrn18*
Transfer RNAs	*trnC-GCA*, *trnD-GTC*, *trnE-TTC*, *trnF-GAA*, *trnH-cp*, *trnI*, *trnK-TTT*, *trnM*, *trnfM-CAT*, *trnN-GTT*, *trnP-TGG*, *trnQ-TTG*, *trnS-GCT*, *trnW-CCA*, *trnY-GTA*

**TABLE 4:** Comparison of some protein coding genes known to be variable between Fabaceae species.

Gene	*Tylosema esculentum*	*Millettia pinnata*	*Vigna radiata*	*Lotus japonicus*	*Glycine max*
*sdh3*	+	+	−	−*	−
*sdh4*	+	−*	−*	−*	−*
*cox2*	+	+	−	+	+
*rpl2*	−	−*	−	−	−
*rpl10*	+	−	−	−*	−
*rps1*	+	+	+	−	+
*rps2*	−	−	−	−	−
*rps7*	−*	−*	−*	−*	−*
*rps10*	+	−	−	+	+
*rps11*	−	−	−	−	−
*rps13*	−	−	−	−	−
*rps19*	−*	−*	−*	−*	−*

+exist and functional, −completely missing, −* present as pseudogene.

## Data Availability Statement

The datasets presented in this study can be found in online repositories. The names of the repository/repositories and accession number(s) can be found below: https://www.ncbi.nlm.nih.gov/genbank/, OK638188 and OK638189; https://www.ncbi.nlm.nih.gov/Traces/study/?acc=PRJNA779273, PRJNA779273.

## Author Contributions

JL carried out the bioinformatics assembly and drafted the manuscript. CC provided extracted DNAs, involved in assembly of mitochondrial genome, and assisted in writing and editing the manuscript. Both authors contributed to the article and approved the submitted version.

## Conflict of Interest

The authors declare that the research was conducted in the absence of any commercial or financial relationships that could be construed as a potential conflict of interest.

## Publisher’s Note

All claims expressed in this article are solely those of the authors and do not necessarily represent those of their affiliated organizations, or those of the publisher, the editors and the reviewers. Any product that may be evaluated in this article, or claim that may be made by its manufacturer, is not guaranteed or endorsed by the publisher.

## Footnotes

1
https://www.ncbi.nlm.nih.gov/sra

2
https://dogma.ccbb.utexas.edu/mitofy/mitofy.cgi

3
https://bigdata.dongguk.edu/gene_project/AGORA/

4
https://chlorobox.mpimp-golm.mpg.de/OGDraw.html

## References

[B1] Adams K. L. Qiu Y. L. Stoutemyer M. Palmer J. D. (2002). Punctuated evolution of mitochondrial gene content: high and variable rates of mitochondrial gene loss and transfer to the nucleus during angiosperm evolution. *Proc. Natl. Acad. Sci. U. S. A.* 99 9905–9912. 10.1073/pnas.042694899 12119382 PMC126597

[B2] Aguiar B. Vieira J. Cunha A. E. Vieira C. P. (2015). No evidence for Fabaceae gametophytic self-incompatibility being determined by Rosaceae, Solanaceae, and Plantaginaceae *S-RNase* lineage genes. *BMC Plant Biol.* 15:129. 10.1186/s12870-015-0497-2 26032621 PMC4451870

[B3] Alverson A. J. Zhuo S. Rice D. W. Sloan D. B. Palmer J. D. (2011). The Mitochondrial genome of the legume *Vigna radiata* and the analysis of recombination across short mitochondrial repeats. *PLoS One* 6:e16404. 10.1371/journal.pone.0016404 21283772 PMC3024419

[B4] Amarteifio J. O. Moholo D. (1998). The chemical composition of four legumes consumed in Botswana. *J. Food Compost. Anal.* 11 329–332. 10.1006/jfca.1998.0595

[B5] Backert S. Nielsen B. L. Börner T. (1997). The mystery of the rings: structure and replication of mitochondrial genomes from higher plants. *Trends Plant Sci.* 2 477–483. 10.1016/s1360-1385(97)01148-5

[B6] Belitz H. D. Grosch W. Schieberle P. (2004). *Food Chemistry.* Berlin: Springer.

[B7] Bower N. Hertel K. Oh J. Storey R. (1988). Nutritional evaluation of marama bean (*Tylosema esculentum*, Fabaceae): analysis of the seed. *Econ. Bot.* 42 533–540. 10.1007/bf02862798

[B8] Chang S. Wang Y. Lu J. Gai J. Li J. Chu P. (2013). The mitochondrial genome of soybean reveals complex genome structures and gene evolution at intercellular and phylogenetic levels. *PLoS One* 8:e56502. 10.1371/annotation/5bf22546-6983-42c9-9cb5-1a6459b29a79 23431381 PMC3576410

[B9] Cheng N. Lo Y. Ansari M. I. Ho K. Jeng S. Lin N. (2016). Correlation between mtDNA complexity and mtDNA replication mode in developing cotyledon mitochondria during mung bean seed germination. *New Phytol.* 213 751–763. 10.1111/nph.14158 27611966

[B10] Claessen H. Keulemans W. Poel B. V. Storme N. D. (2019). Finding a compatible partner: self-incompatibility in European pear (*Pyrus communis*); molecular control, genetic determination, and impact on fertilization and fruit set. *Front. Plant Sci.* 10:407. 10.3389/fpls.2019.00407 31057563 PMC6477101

[B11] Cullis C. Chimwamurombe P. Barker N. Kunert K. Vorster J. (2018). Orphan legumes growing in dry environments: marama bean as a case study. *Front. Plant Sci.* 9:1199. 10.3389/fpls.2018.01199 30158948 PMC6104163

[B12] Dakora F. D. (2013). Biogeographic distribution, nodulation and nutritional attributes of underutilized indigenous African legumes. *Acta Hortic.* 979 53–64. 10.17660/actahortic.2013.979.3

[B13] Epstein C. B. Waddle J. A. Hale W. Davé V. Thornton J. Macatee T. L. (2001). Genome-wide responses to mitochondrial dysfunction. *Mol. Biol. Cell* 12 297–308. 10.1091/mbc.12.2.297 11179416 PMC30944

[B14] Fauron C. Casper M. Gao Y. Moore B. (1995). The maize mitochondrial genome: dynamic, yet functional. *Trends Genet.* 11 228–235. 10.1016/s0168-9525(00)89056-3 7638905

[B15] Galluzzi L. Kepp O. Trojel-Hansen C. Kroemer G. (2012). Mitochondrial control of cellular life, stress, and death. *Circ. Res.* 111 1198–1207. 10.1161/circresaha.112.268946 23065343

[B16] García-Valencia L. E. Bravo-Alberto C. E. Wu H. M. Rodríguez-Sotres R. Cheung A. Y. Cruz-García F. (2017). SIPP, a novel mitochondrial phosphate carrier, mediates in self-incompatibility. *Plant Physiol.* 175 1105–1120. 10.1104/pp.16.01884 28874520 PMC5664454

[B17] Jackson J. C. Duodu K. G. Holse M. Lima de Faria M. D. Jordaan D. Chingwaru W. (2010). The morama bean (*Tylosema esculentum*): a potential crop for southern Africa. *Adv. Food Nutr. Res.* 61 187–246. 10.1016/B978-0-12-374468-5.00005-2 21092905

[B18] Kazakoff S. H. Imelfort M. Edwards D. Koehorst J. Biswas B. Batley J. (2012). Capturing the biofuel wellhead and powerhouse: the chloroplast and mitochondrial genomes of the leguminous feedstock tree *Pongamia pinnata*. *PLoS One* 7:e51687. 10.1371/journal.pone.0051687 23272141 PMC3522722

[B19] Kempken F. Pring D. R. (1999). Male sterility in higher plants - fundamentals and applications. *Prog. Bot.* 60 139–166.

[B20] Kim Y. Cullis C. (2017). A novel inversion in the chloroplast genome of marama (*Tylosema esculentum*). *J. Exp. Bot.* 68 2065–2072. 10.1093/jxb/erw500 28158587 PMC5429017

[B21] Kozik A. Rowan B. A. Lavelle D. Berke L. Schranz M. E. Michelmore R. W. (2019). The alternative reality of plant mitochondrial DNA: one ring does not rule them all. *PLoS Genet.* 15:e1008373. 10.1371/journal.pgen.1008373 31469821 PMC6742443

[B22] Kubo T. Mikami T. (2007). Organization and variation of angiosperm mitochondrial genome. *Physiol. Plant.* 129 6–13. 10.1111/j.1399-3054.2006.00768.x

[B23] Maréchal A. Brisson N. (2010). Recombination and the maintenance of plant organelle genome stability. *New Phytol.* 186 299–317. 10.1111/j.1469-8137.2010.03195.x 20180912

[B24] Miller A. J. Gross B. L. (2011). From forest to field: perennial fruit crop domestication. *Am. J. Bot.* 98 1389–1414. 10.3732/ajb.1000522 21865506

[B25] Mower J. P. Sloan D. B. Alverson A. J. (2012). Plant mitochondrial genome diversity: the genomics revolution. *Plant Genome Divers.* 1 123–144. 10.1007/978-3-7091-1130-7_9

[B26] National Research Council [NRC] (2006). *Lost Crops of Africa: Volume II: Vegetables.* Washington: National Academies Press.

[B27] Negruk V. (2013). Mitochondrial genome sequence of the legume *Vicia faba*. *Front. Plant Sci.* 4:128. 10.3389/fpls.2013.00128 23675376 PMC3646248

[B28] Nepolo E. (2010). *Assessment of genetic variations within and between populations of marama bean (Tylosema Esculentum (Burchell) Schreiber) based on microsatellites (SSRs) and intergenic spacer length variation markers in the Namibian germplasm.* Windhoek: University of Namibia.

[B29] Palmer J. D. Herbon L. A. (1988). Plant mitochondrial DNA evolved rapidly in structure, but slowly in sequence. *J. Mol. Evol.* 28 87–97. 10.1007/bf02143500 3148746

[B30] Siekevitz P. (1957). Powerhouse of the cell. *Sci. Am.* 197 131–140. 10.1038/scientificamerican0757-131

[B31] Sloan D. B. Alverson A. J. Chuckalovcak J. P. Wu M. McCauley D. E. Palmer J. D. (2012). Rapid evolution of enormous, multichromosomal genomes in flowering plant mitochondria with exceptionally high mutation rates. *PLoS Biol.* 10:e1001241. 10.1371/journal.pbio.1001241 22272183 PMC3260318

[B32] Takundwa M. Chimwamurombe P. M. Cullis C. A. (2012). A chromosome count in marama bean (*Tylosema esculentum*) by Feulgen staining using garden pea (*Pisum sativum* L.) as a standard. *Res. J. Biol.* 2 177–181.

[B33] Unseld M. Marienfeld J. R. Brandt P. Brennicke A. (1997). The mitochondrial genome of *Arabidopsis thaliana* contains 57 genes in 366,924 nucleotides. *Nat. Genet.* 15, 57–61. 10.1038/ng0197-57 8988169

[B34] van der Maesen L. J. G. (2006). “Tylosema esculentum,” in *PROTA*, eds Brink M. Belay G. (Netherlands: Earthprint Ltd).

[B35] Vietmeyer N. D. (1986). Lesser-known plants of potential use in agriculture and forestry. *Science* 232 1379–1384. 10.1126/science.232.4756.1379 17828913

[B36] Wallace D. C. (2005). A mitochondrial paradigm of metabolic and degenerative diseases, aging, and cancer: a dawn for evolutionary biology. *Annu. Rev. Genet.* 39 359–407. 10.1146/annurev.genet.39.110304.095751 16285865 PMC2821041

