# Supplementary material for: The Multipartite Mitochondrial Genome of Marama (Tylosema esculentum)
Source: Front Plant Sci. 2021 Dec 8;12:787443. doi: 10.3389/fpls.2021.787443 (PMC8692981; doi:10.3389/fpls.2021.787443)
Supplement: Supplementary file 1 [file Data_Sheet_1.PDF]

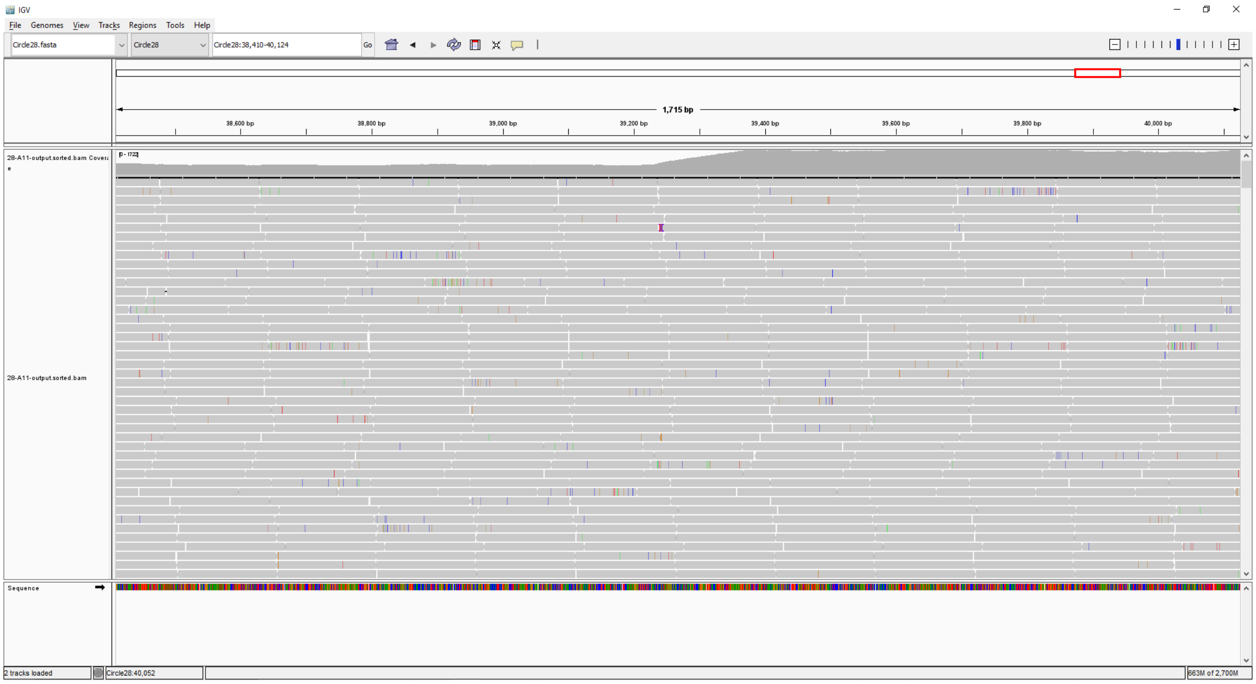

Figure S1. IGV visualization of the long repeat shared by circle 28 (M2) and circle LS1a2 (M1) shows the coverage is doubled. The Illumina raw reads of individual A11 were aligned to Circle 28 using Bowtie 2 and the result was visualized in IGV after converting the sam file to a sorted bam file by Samtools 1.7. The number of reads increased from 700 to 1600 after 39,200, since this region was shared by both the molecules circle 28 and LS1a2.

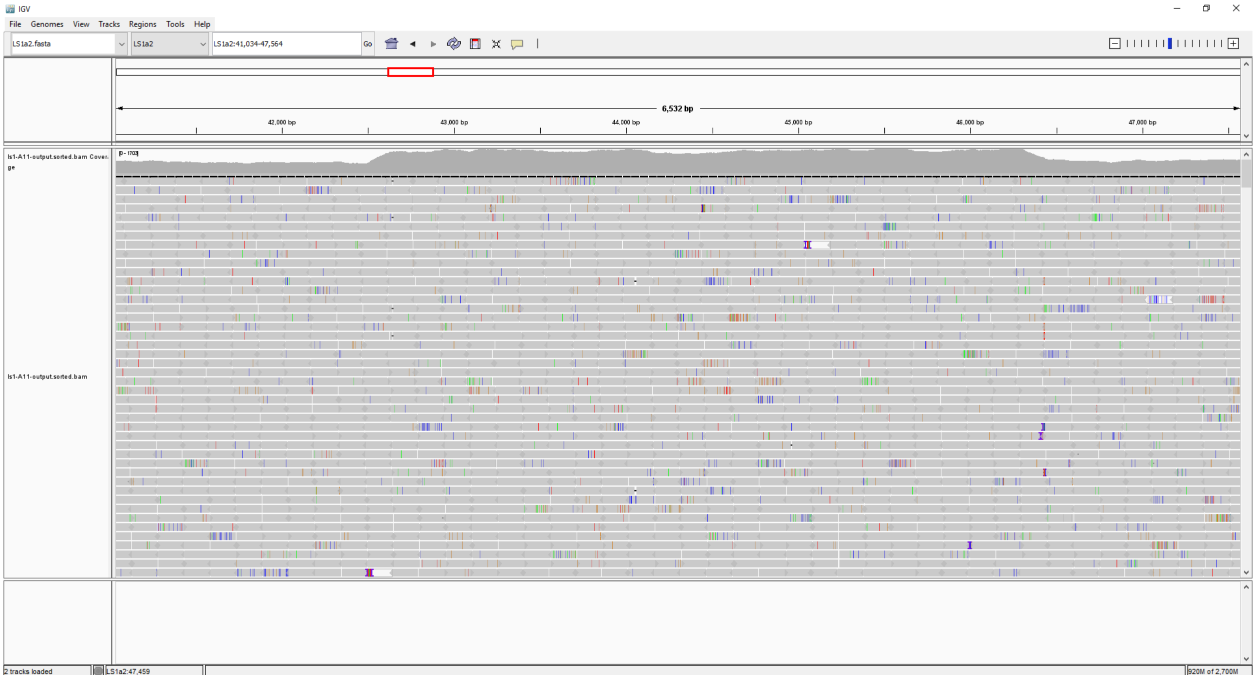

Figure S2. IGV visualization of the long repeat shared by circle LS1a2 (M1) and circle 313 (M3) displays an increase of the coverage at this region. The Illumina raw reads of individual A11 were aligned to Circle LS1a2 using Bowtie 2 and the result was visualized in IGV after

converting the sam file to a sorted bam file by Samtools 1.7. The read count increased from 750 to 1500 for the sequence between 42,500 and 46,500 where the overlap between circle LS1a2 and circle 313 located.

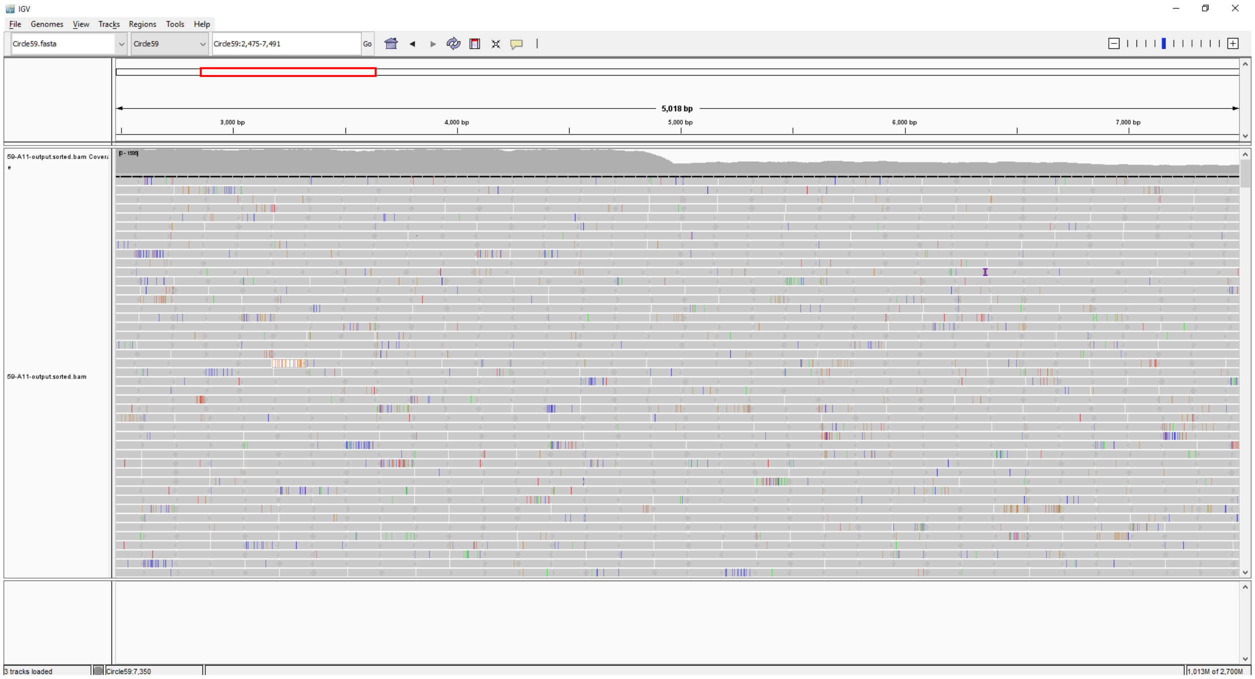

Figure S3. IGV visualization of the long repeat shared by circle 59 (M4) and circle 192 (M5) shows an increase of the coverage at the overlap. The Illumina raw reads of individual A11 were aligned to Circle 59 using Bowtie 2 and the result was visualized in IGV after converting the sam file to a sorted bam file by Samtools 1.7. The depth was about 1,500 (two copies) for the sequence before 5,000, since this piece represented the repeat shared by both the molecules circle 59 and circle 192. However, the sequence after 5,000 was exclusive to circle 59, so the number of reads decreased to one copy 750.

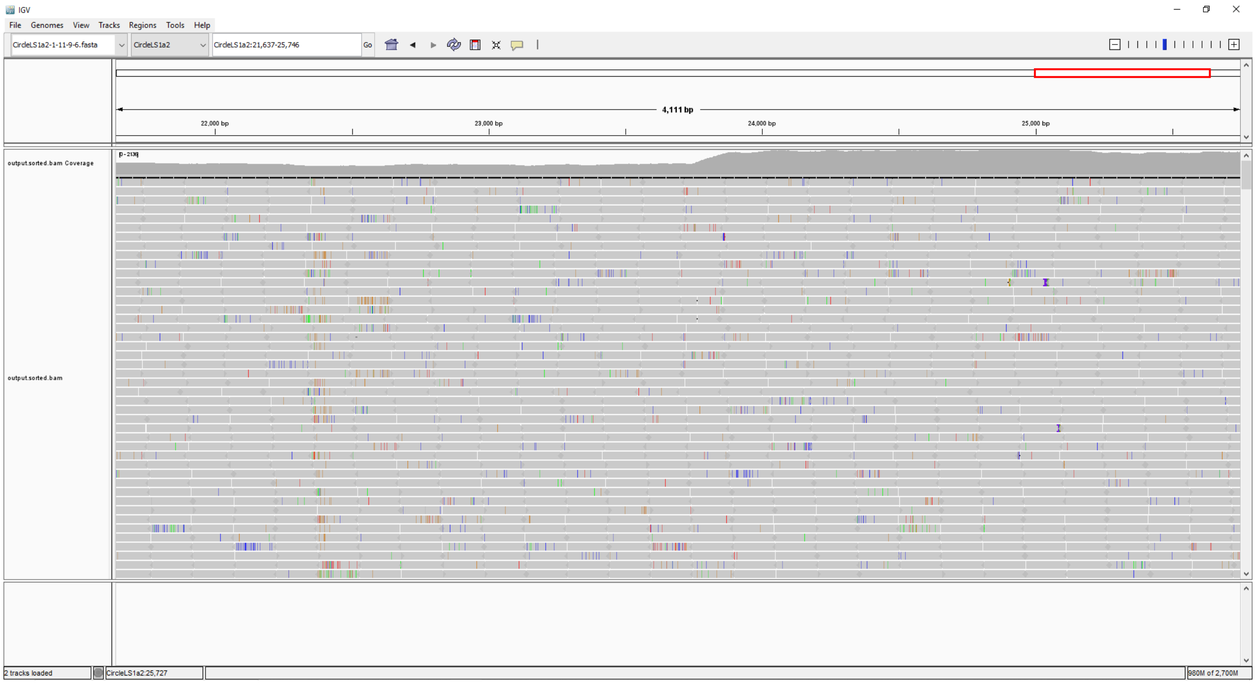

Figure S4. IGV visualization of a part of the 2,351bp inverted repeat on LS1a2 (M1) showing a doubled coverage. The Illumina raw reads of individual A11 were aligned to a part of the circle LS1a2 (26kb fragment) using Bowtie 2 and the result was visualized in IGV after converting the sam file to a sorted bam file by Samtools 1.7. The depth of the sequence after 23,750 (where the repeat located) increased from 800 to 1,800.

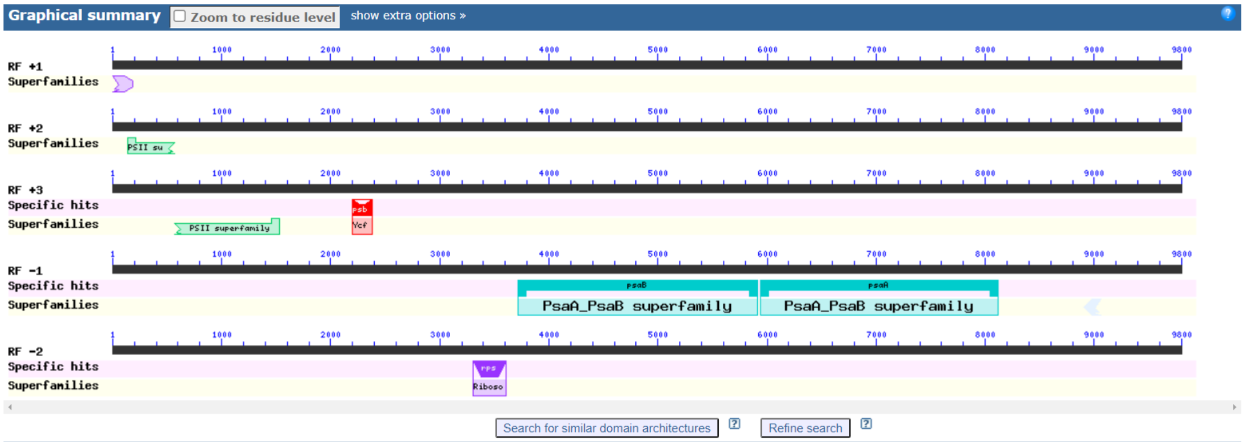

List of domain hits

Name	Accession	Description	Interval	E-value
[+] Photo_RC super family	cl08220	D1, D2 subunits of photosystem II (PSII); M, L subunits of bacterial photosynthetic reaction ...	4-183	8.71e-37
[+] PSII super family	cl08223	Photosystem II protein;	134-568	1.13e-89
[+] PSII super family	cl08223	Photosystem II protein;	570-1526	0e+00
[+] psbZ	CHL00082	photosystem II protein Z	2199-2384	6.42e-19
[+] psaB	CHL00054	photosystem I P700 chlorophyll a apoprotein A2	3715-5907	0e+00
[+] psaA	CHL00056	photosystem I P700 chlorophyll a apoprotein A1	5941-8115	0e+00
[+] ycf3 super family	cl31773	photosystem I assembly protein Ycf3	8899-9057	1.24e-26
[+] rps14	CHL00074	ribosomal protein S14	3309-3608	1.94e-53

Figure S5. Conserved domains found in the 9,798 bp chloroplast mitochondrial shared DNA sequence in marama. Conserved domains were searched on the long fragment shared by marama chloroplast and mitochondrial DNA on NCBI

(https://www.ncbi.nlm.nih.gov/Structure/cdd/wrpsb.cgi). Full length chloroplast gene *psaA*, *psaB* and part of *psbC* and *rps14* were found on the sequence.

12 contigs were got from the assembly of PacBio reads using Canu v2.2. All the contigs were aligned to the structure units of LS1 and LS2 independently to confirm the existence of the structure. The complete assembly was not obtained from Canu directly due to the insufficient read coverage at the junctions of the contigs, which have been verified by the genome assembly on Illumina reads.

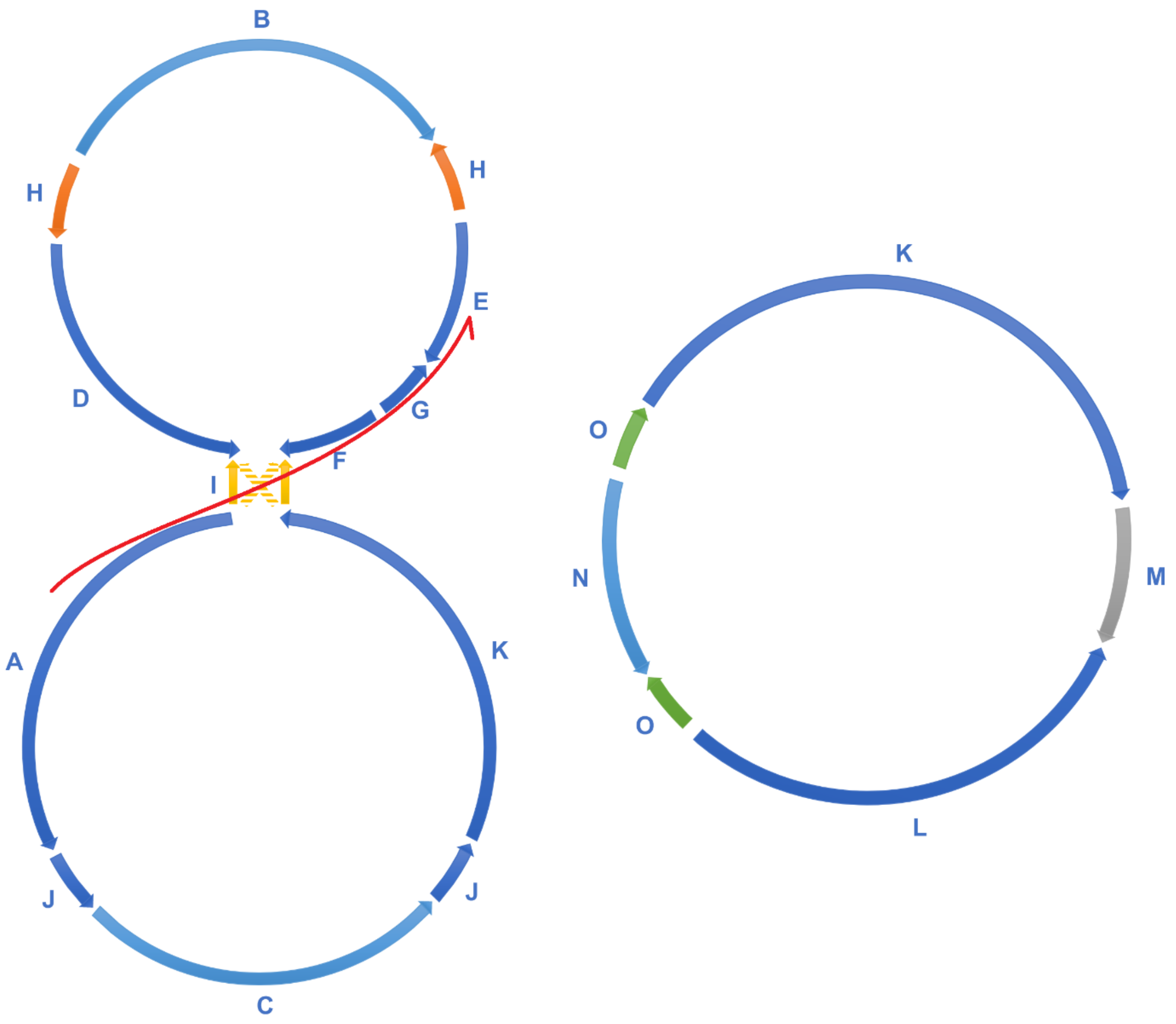

Figure S6. The mitochondrial contig obtained from Canu verified the connection of some structural units. Read 78 with a length of 21,293 bp was got from the direct assembly of the 1.78

Gb PacBio reads by Canu 2.2 (GenomeSize =20m, correctedErrorRate=0.15). The sequence was BLASTed with the structural units on NCBI, and a red curve on the mtDNA structure diagram was used to show its position.

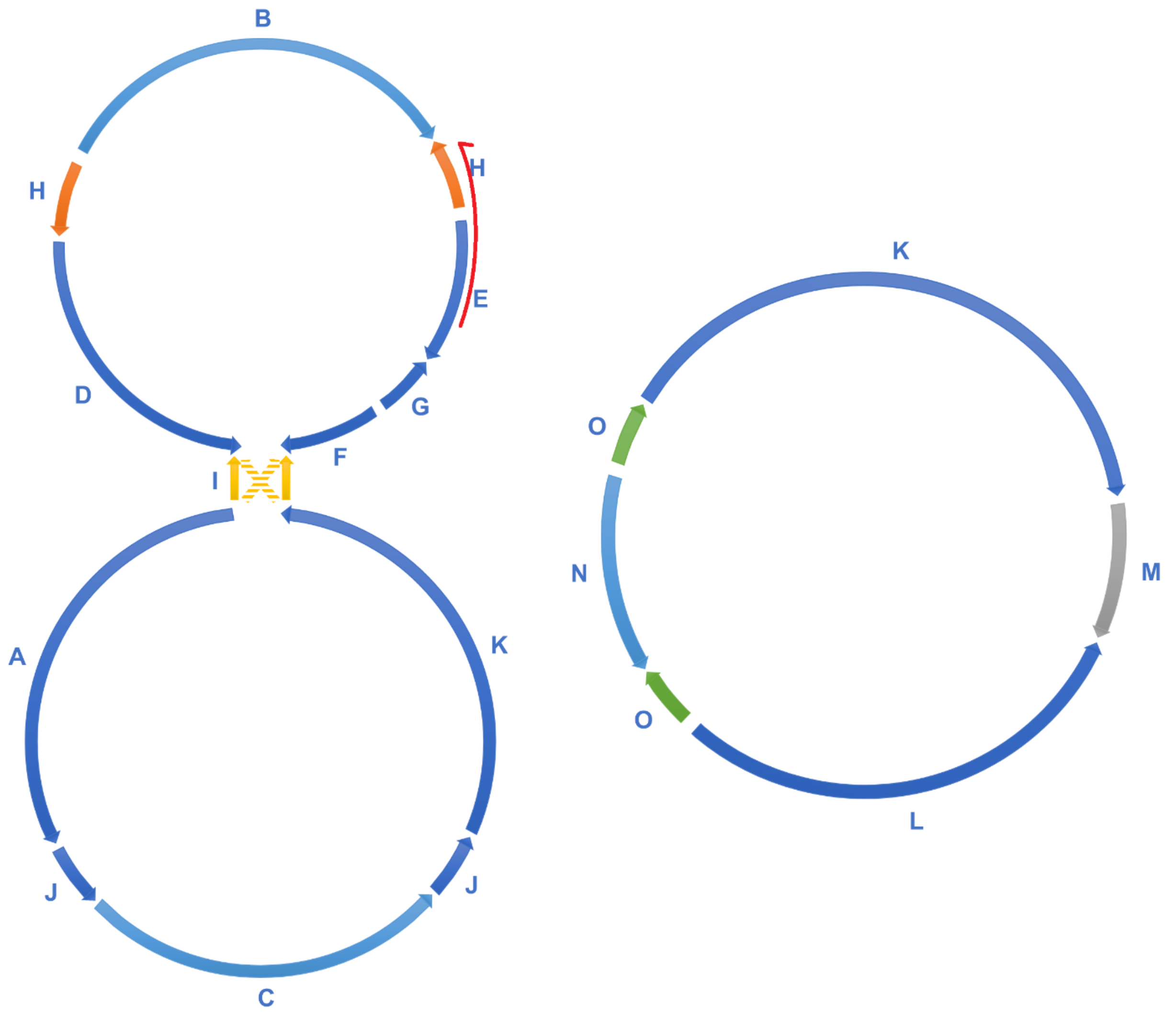

Figure S7. The mitochondrial contig obtained from Canu verified the connection of some structural units. Read 117 with a length of 25,771 bp was got from the direct assembly of the 1.78 Gb PacBio reads by Canu 2.2 (GenomeSize =20m, correctedErrorRate=0.15). The sequence was BLASTed with the structural units on NCBI, and a red curve on the mtDNA structure diagram was used to show its position.

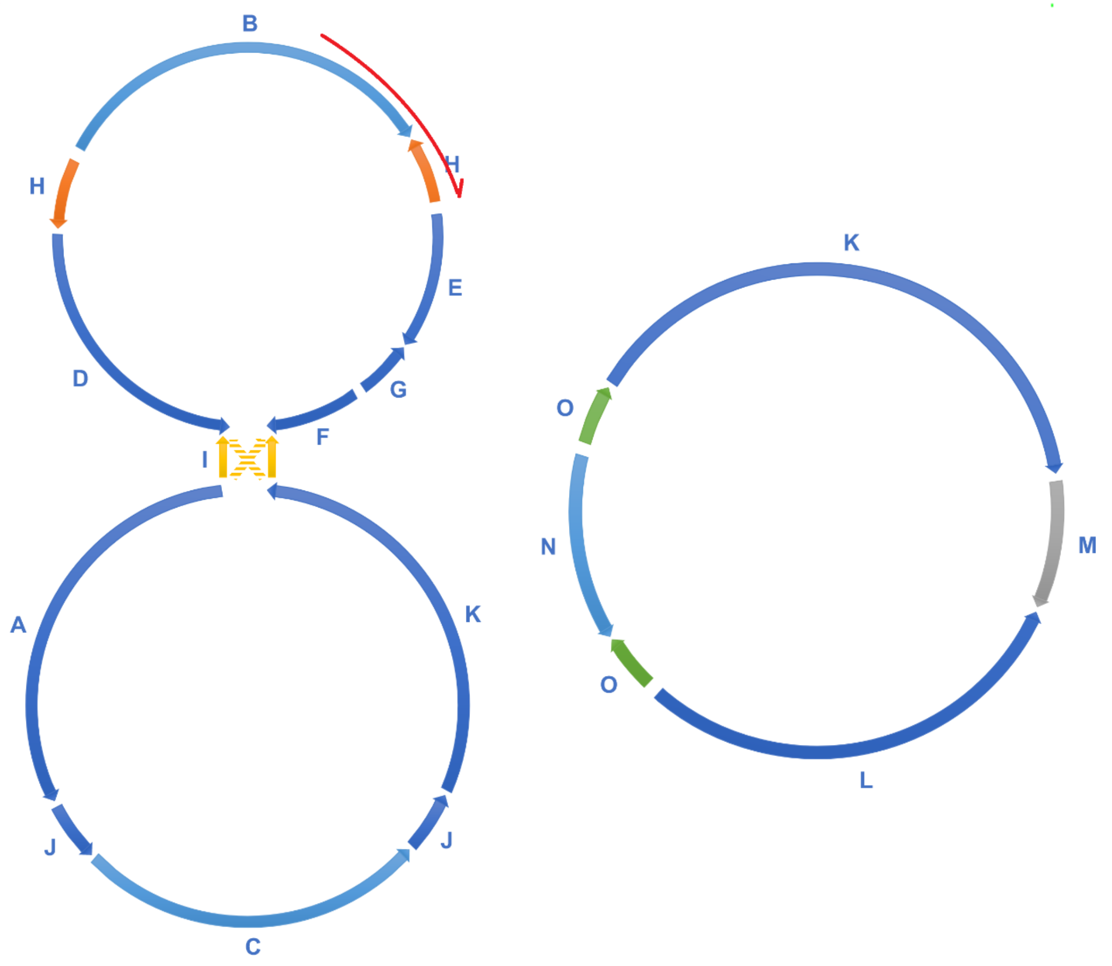

Figure S8. The mitochondrial contig obtained from Canu verified the connection of some structural units. Read 57 with a length of 18,913 bp was got from the direct assembly of the 1.78 Gb PacBio reads by Canu 2.2 (GenomeSize =20m, correctedErrorRate=0.15). The sequence was BLASTed with the structural units on NCBI, and a red curve on the mtDNA structure diagram was used to show its position.

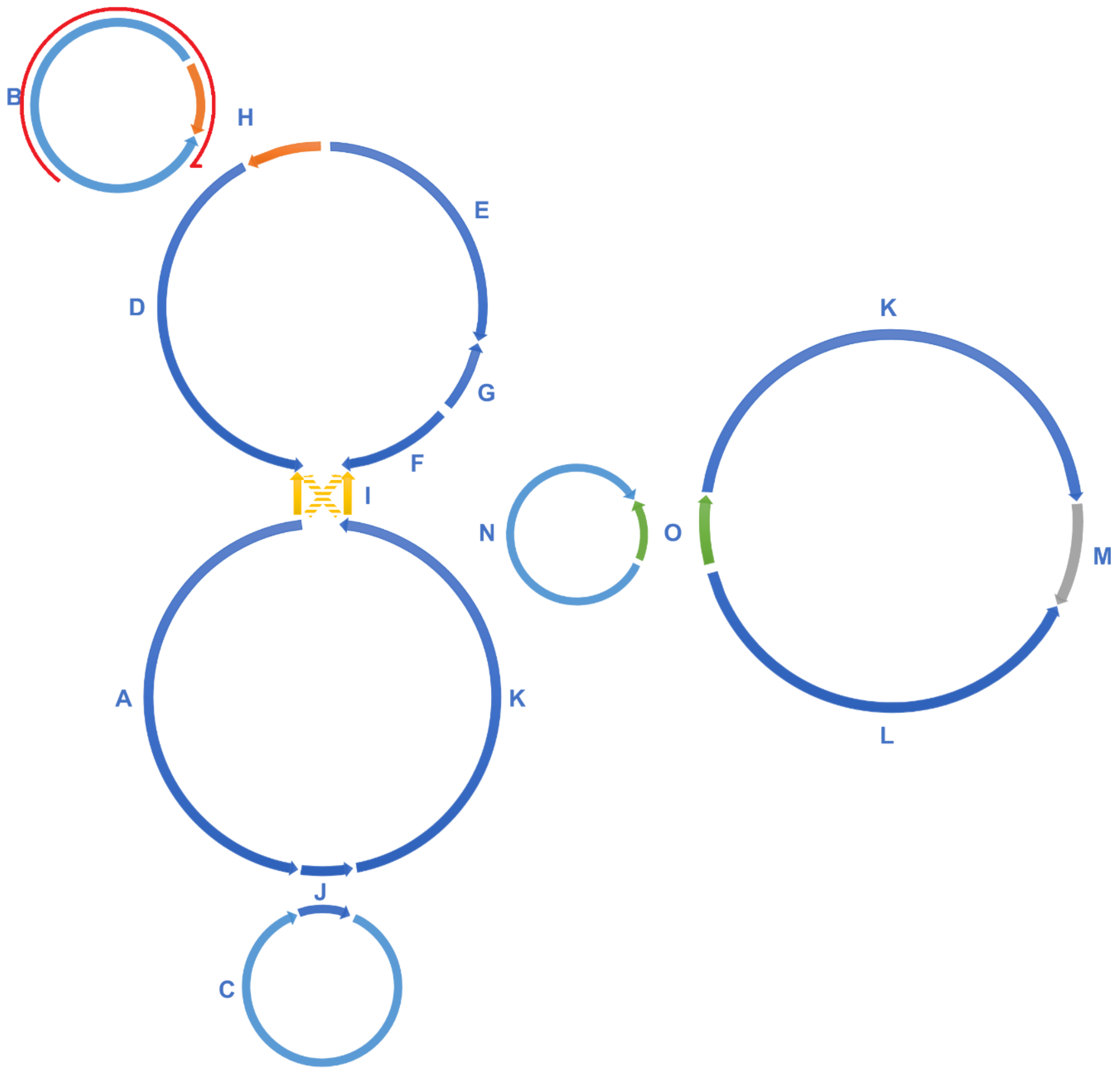

Figure S9. The mitochondrial contig obtained from Canu verified the connection of some structural units. Read 111 with a length of 29,553 bp was got from the direct assembly of the 1.78 Gb PacBio reads by Canu 2.2 (GenomeSize =20m, correctedErrorRate=0.15). The sequence was BLASTed with the structural units on NCBI, and a red curve on the mtDNA structure diagram was used to show its position.

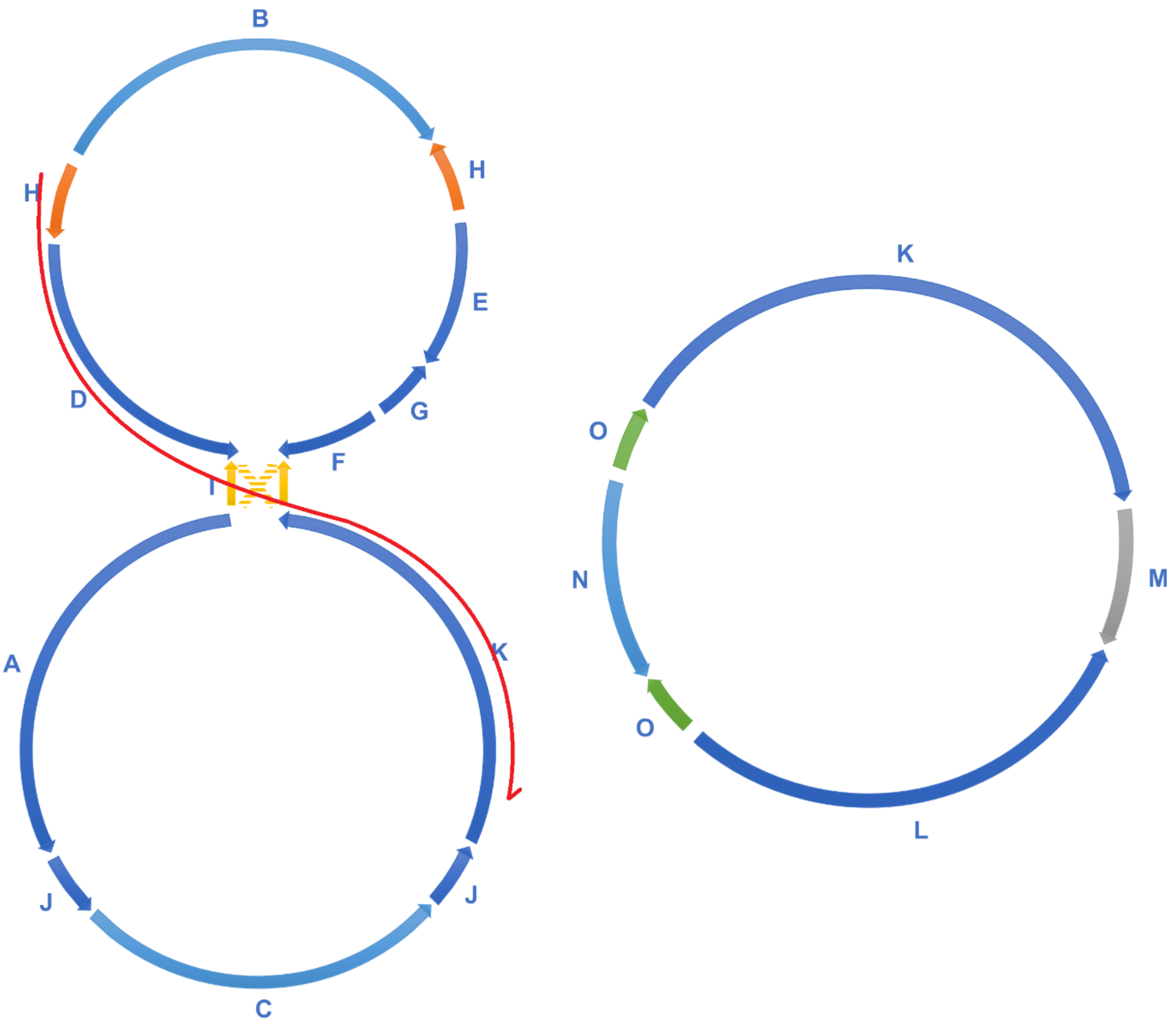

Figure S10. The mitochondrial contig obtained from Canu verified the connection of some structural units. Read 297 with a length of 70,055 bp was got from the direct assembly of the 1.78 Gb PacBio reads by Canu 2.2 (GenomeSize =20m, correctedErrorRate=0.15). The sequence was BLASTed with the structural units on NCBI, and a red curve on the mtDNA structure diagram was used to show its position.

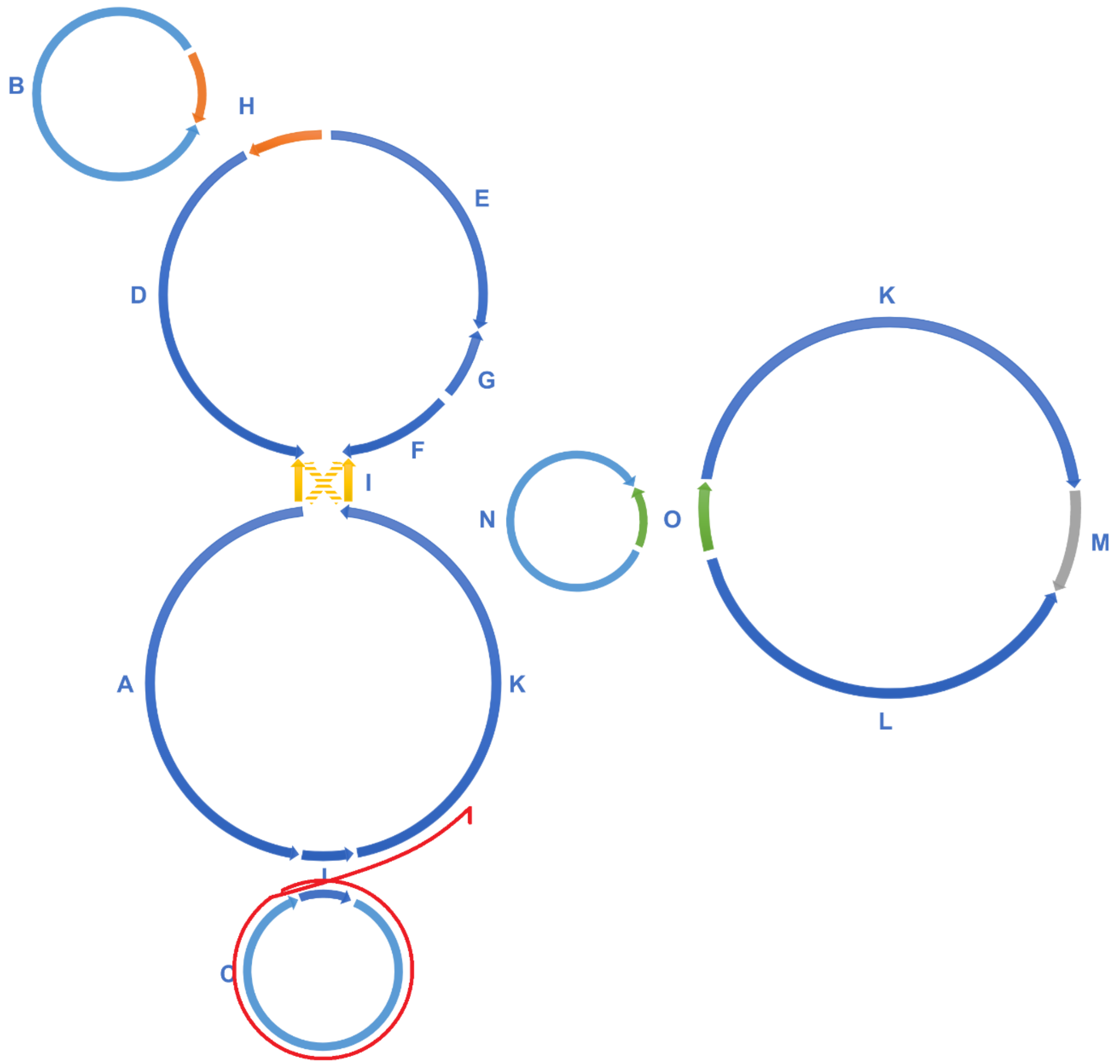

Figure S11. The mitochondrial contig obtained from Canu verified the connection of some structural units. Read 186 with a length of 55,037 bp was got from the direct assembly of the 1.78 Gb PacBio reads by Canu 2.2 (GenomeSize =20m, correctedErrorRate=0.15). The sequence was BLASTed with the structural units on NCBI, and a red curve on the mtDNA structure diagram was used to show its position.

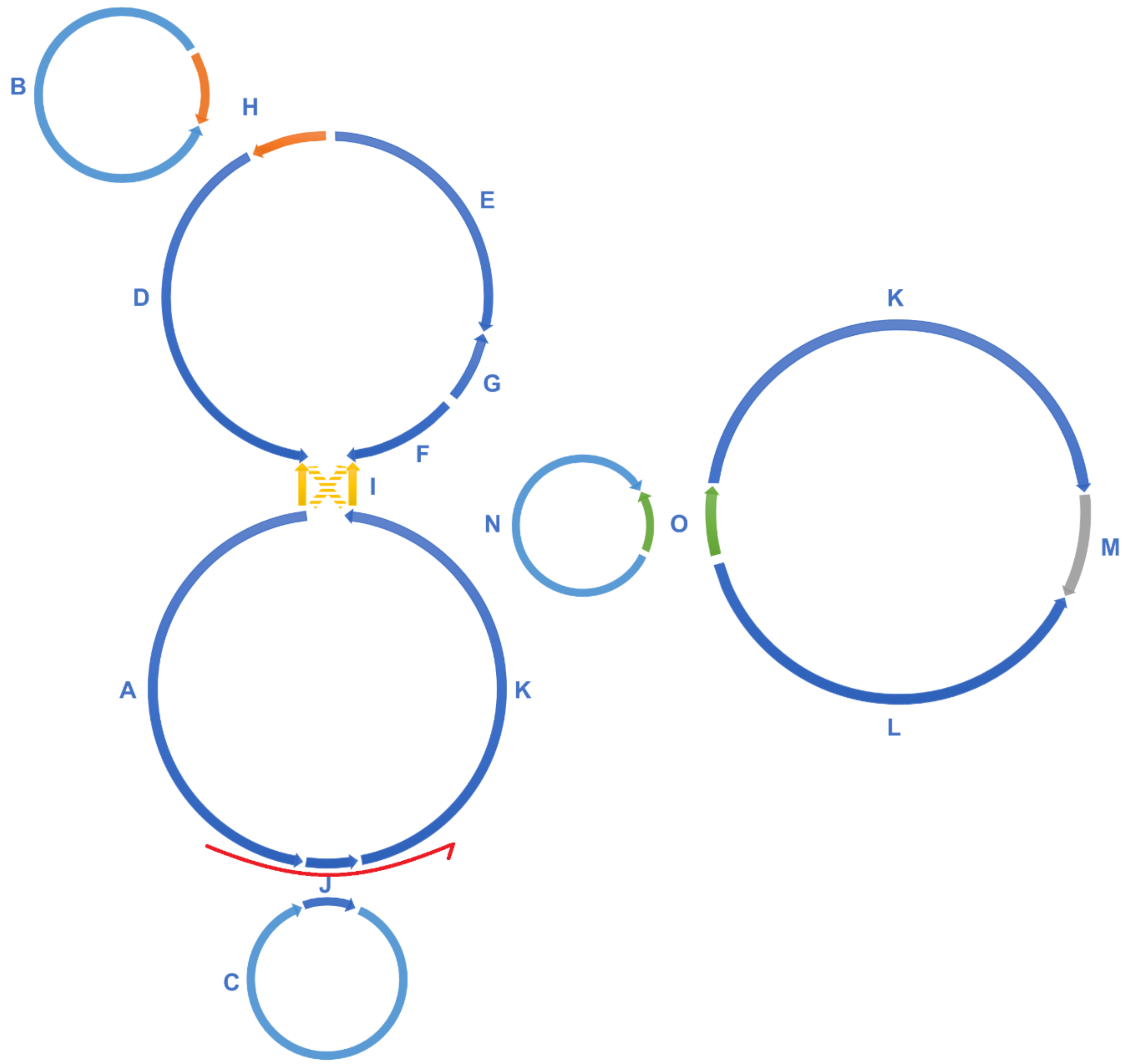

Figure S12. The mitochondrial contig obtained from Canu verified the connection of some structural units. Read 22 with a length of 14,304 bp was got from the direct assembly of the 1.78 Gb PacBio reads by Canu 2.2 (GenomeSize =20m, correctedErrorRate=0.15). The sequence was BLASTed with the structural units on NCBI, and a red curve on the mtDNA structure diagram was used to show its position.

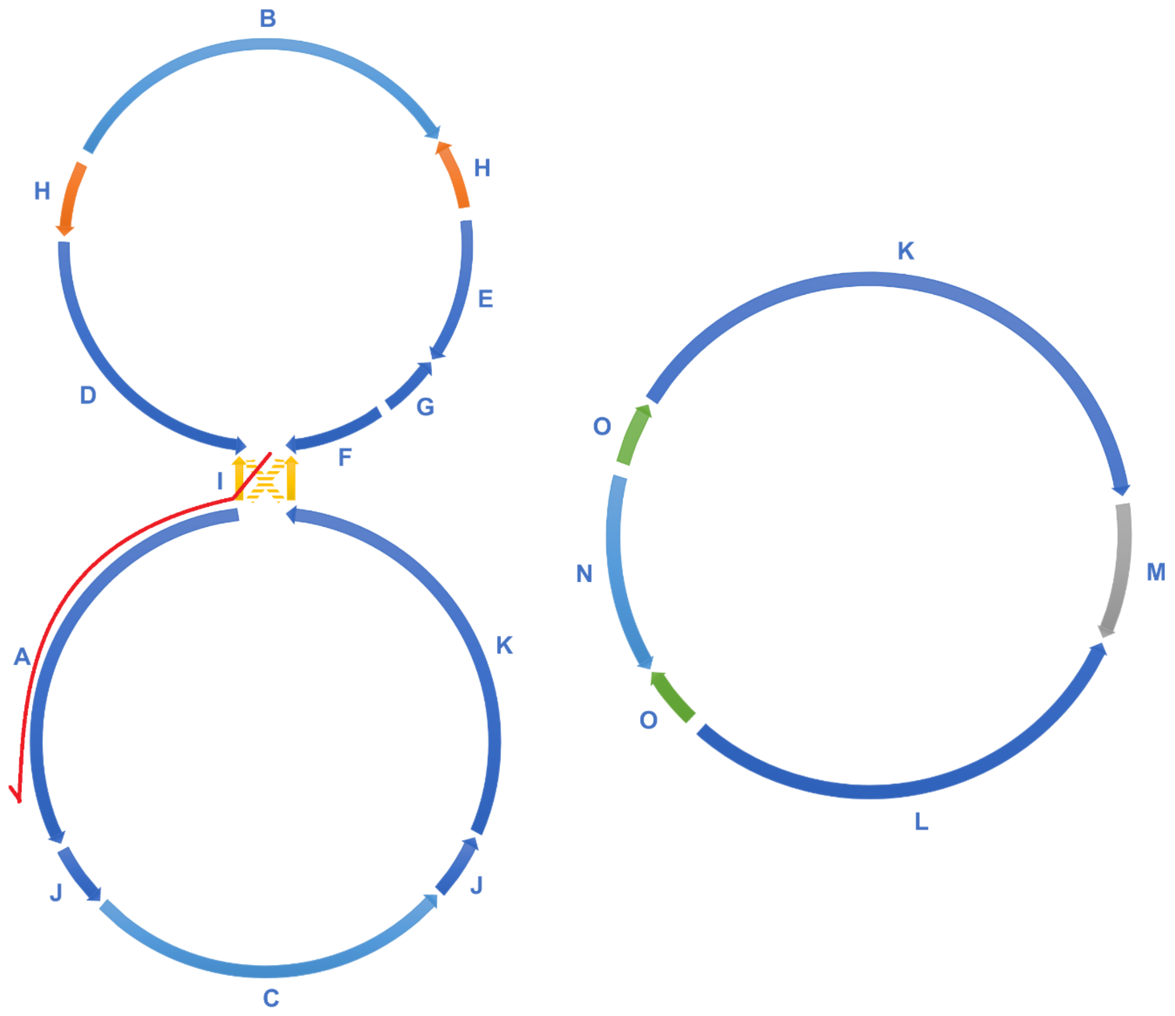

Figure S13. The mitochondrial contig obtained from Canu verified the connection of some structural units. Read 130 with a length of 31,102 bp was got from the direct assembly of the 1.78 Gb PacBio reads by Canu 2.2 (GenomeSize =20m, correctedErrorRate=0.15). The sequence was BLASTed with the structural units on NCBI, and a red curve on the mtDNA structure diagram was used to show its position.

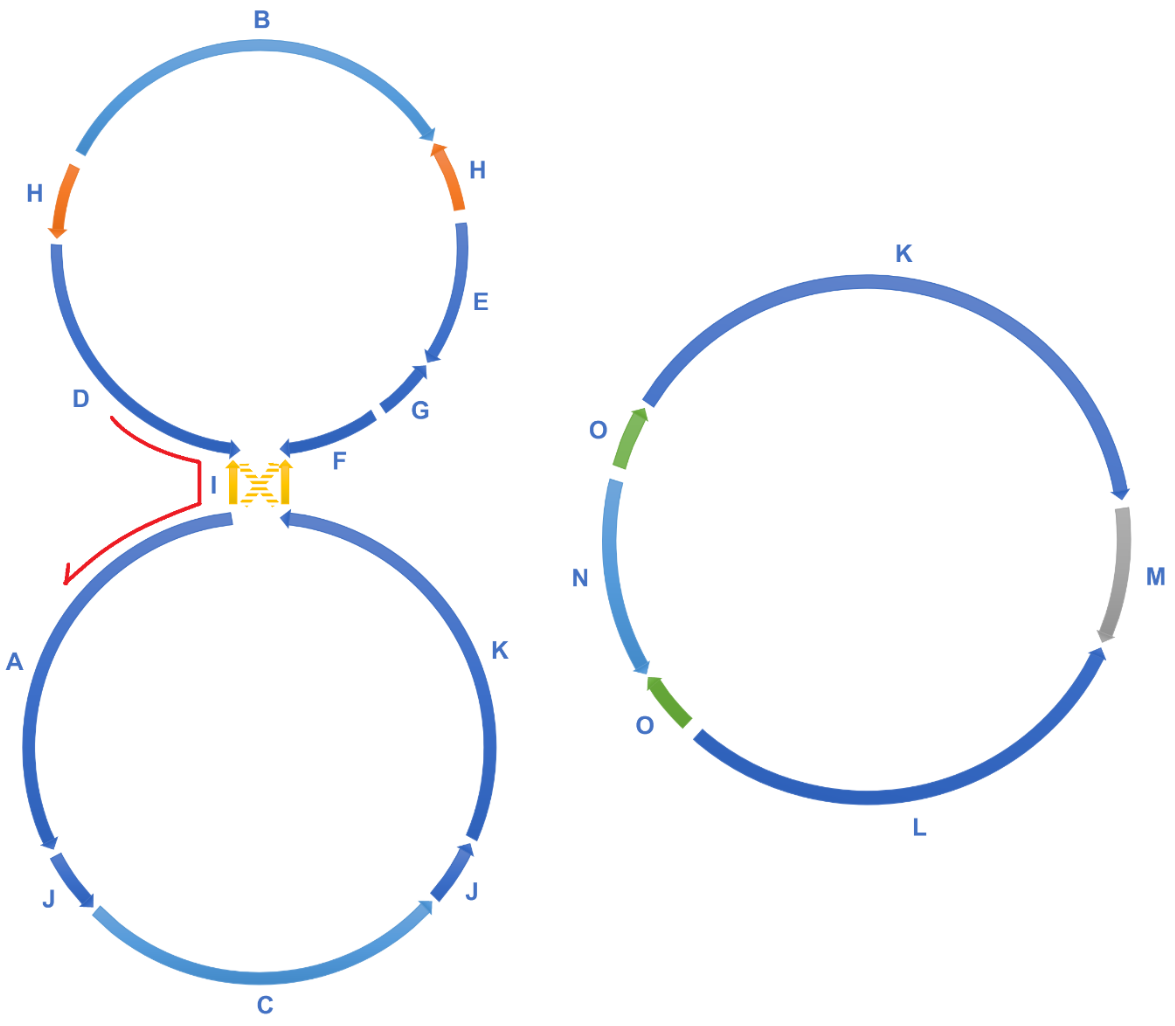

Figure S14. The mitochondrial contig obtained from Canu verified the connection of some structural units. Read 4 with a length of 7,143 bp was got from the direct assembly of the 1.78 Gb PacBio reads by Canu 2.2 (GenomeSize =20m, correctedErrorRate=0.15). The sequence was BLASTed with the structural units on NCBI, and a red curve on the mtDNA structure diagram was used to show its position.

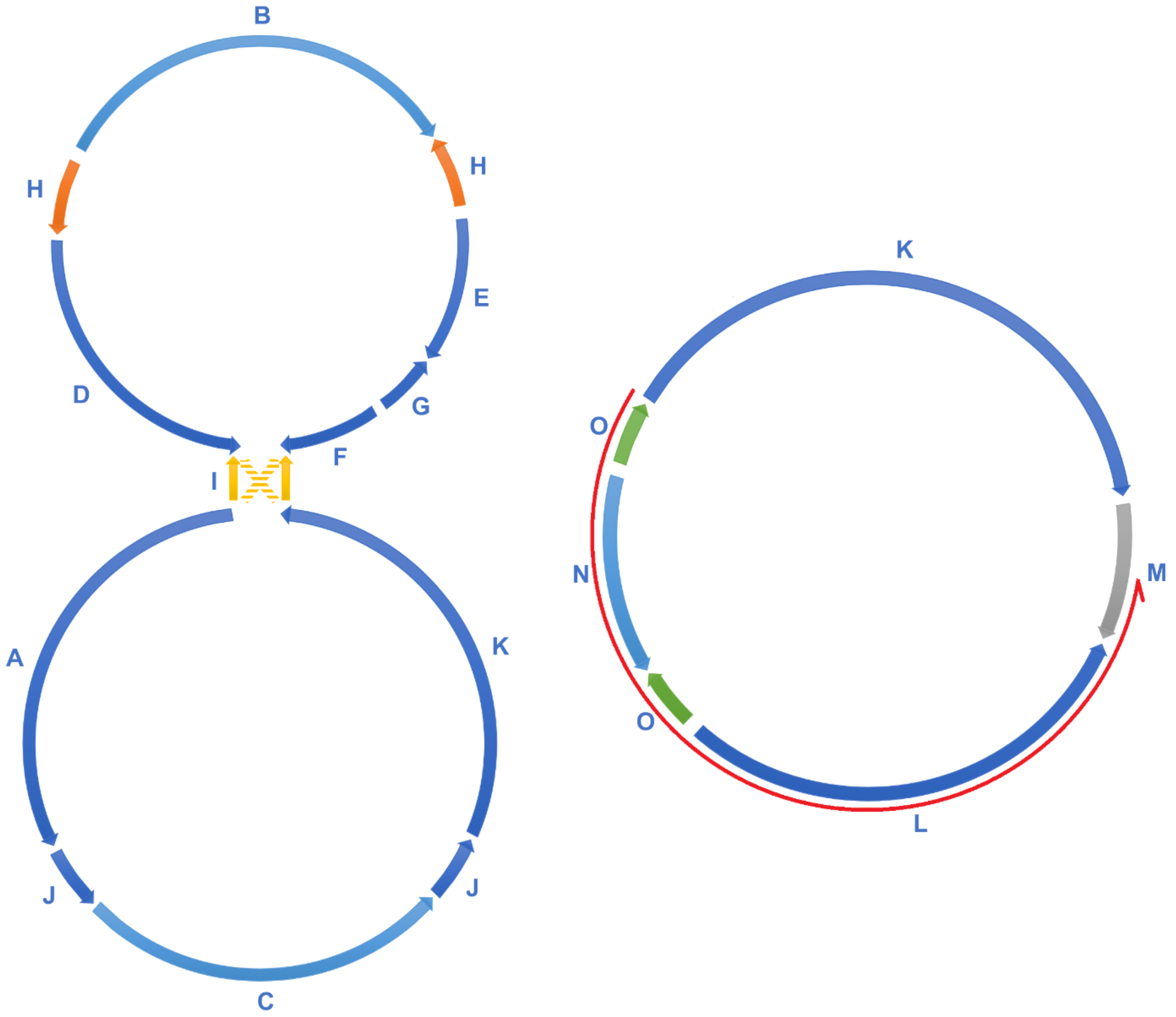

Figure S15. The mitochondrial contig obtained from Canu verified the connection of some structural units. Read 371 with a length of 84,053 bp was got from the direct assembly of the 1.78 Gb PacBio reads by Canu 2.2 (GenomeSize =20m, correctedErrorRate=0.15). The sequence was BLASTed with the structural units on NCBI, and a red curve on the mtDNA structure diagram was used to show its position.

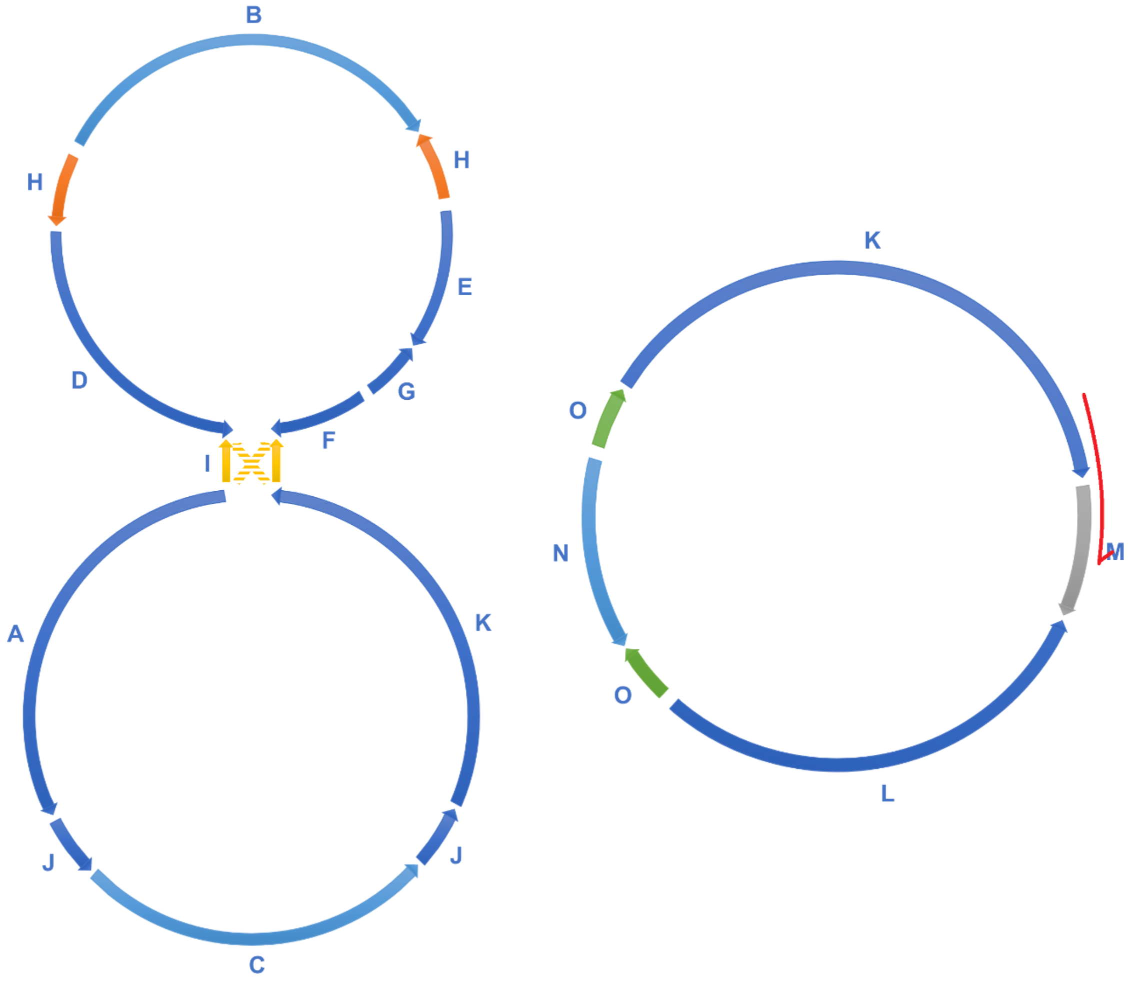

Figure S16. The mitochondrial contig obtained from Canu verified the connection of some structural units. Read 53 with a length of 11,701 bp was got from the direct assembly of the 1.78 Gb PacBio reads by Canu 2.2 (GenomeSize =20m, correctedErrorRate=0.15). The sequence was BLASTed with the structural units on NCBI, and a red curve on the mtDNA structure diagram was used to show its position.

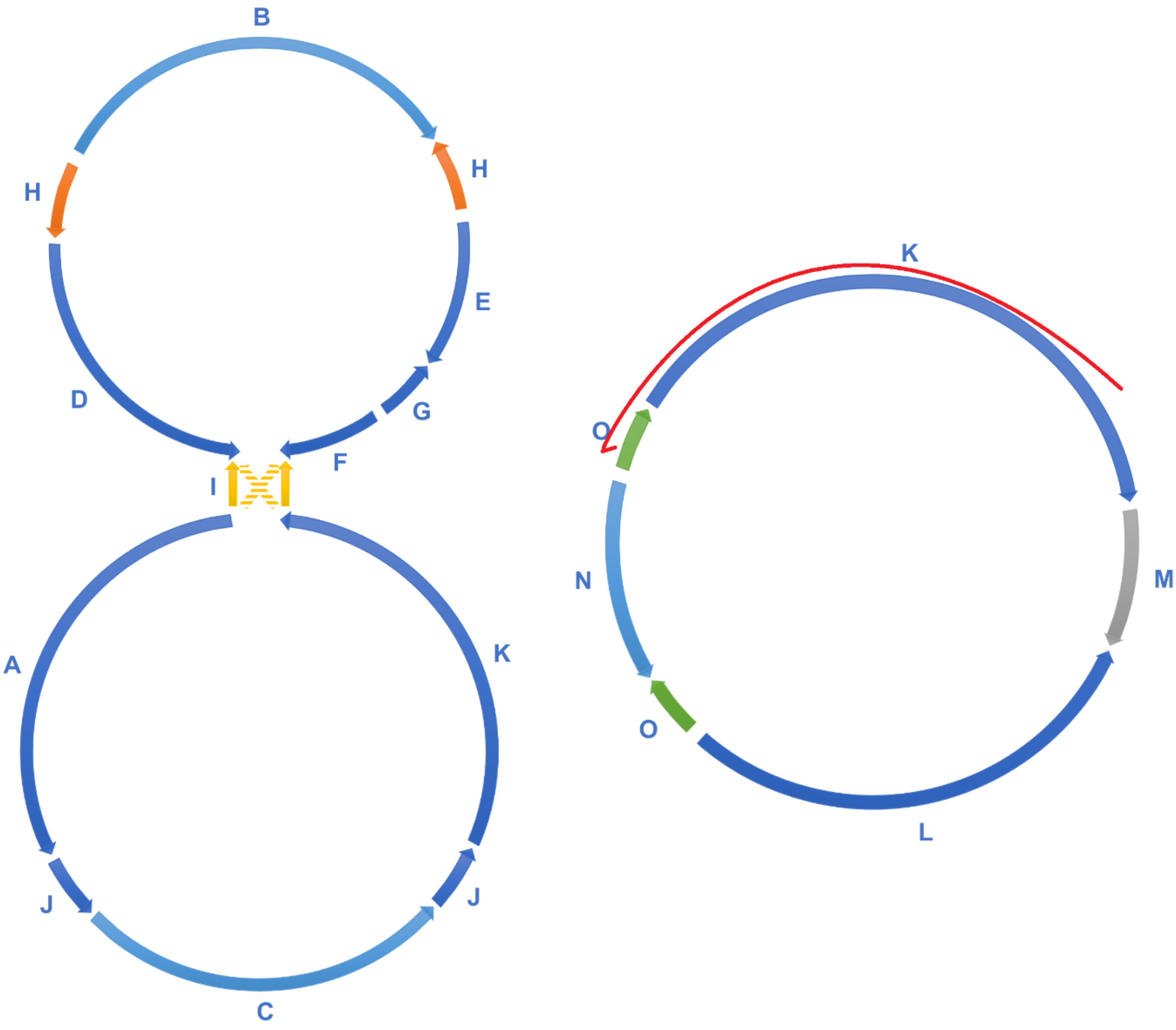

Figure S17. The mitochondrial contig obtained from Canu verified the connection of some structural units. Read 231 with a length of 53,705 bp was got from the direct assembly of the 1.78 Gb PacBio reads by Canu 2.2 (GenomeSize =20m, correctedErrorRate=0.15). The sequence was BLASTed with the structural units on NCBI, and a red curve on the mtDNA structure diagram was used to show its position.

```
ORF47       MEFSPRAAELTTLLESRIKGFYTHFQVDEIGRVVSVGDGIARVYGLNEIQAGEMVEFASG        60
ORF105      MEFSPRAAELTTLLESRIKGFYTHFQVDEIGRVVSVGDGIARVYGLNEIQAGEMVEFASG        60
            ************************************************************

ORF47       VKGIALNLENENVGIVVFGSDTAIKEGDLVKRTGSIVDVPAGKAMLGRVVDALGVPIDGR        120
ORF105      VKGIALNLENENVGIVVFGSDTAIKEGDLVKRTGSIVDVPAGKAMLGRVVDALGVPIDGR        120
            ************************************************************

ORF47       GALSDHERRRVEVKAPGIIERKSVHEPMQTGLKAVDSLVPIGRGQRELIIGDRQTGKTAI        180
ORF105      GALSDHERRRVEVKAPGIIERKSVHEPMQTGLKAVDSLVPIGRGQRELIIGDRQTGKTAI        180
            ************************************************************

ORF47       AIDTILNQKQMNSSSTSDSETLYCVYVAIGQKRSTVAQLVQILSEANALEYSILVAATAS        240
ORF105      AIDTILNQKQMNSSSTSDSETLYCVYVAIGQKRSTVAQLVQILSEANALEYSILVAATAS        240
            ************************************************************

ORF47       DPAPLQFLAPYSGCAMGEYFRDNGMHALIIYDDLSKQAVAYRQMSLLLRRPPGREAFPGD        300
ORF105      DPAPLQFLAPYSGCAMGEYFRDNGMHALIIYDDLSKQAVAYRQMSLLLRRPPGREAFPGD        300
            ************************************************************

ORF47       VFYLHSRLLERAAKRSDQTGAGSLTALPVIETQAGDVSAYIPTNVIPITDGQICSETELF        360
ORF105      VFYLHSRLLERAAKRSDQTGAGSLTALPVIETQAGDVSAYIPTNVIPITDGQICSETELF        360
            ************************************************************

ORF47       YRGIRPAINVGLSVSRVGSAAQLKSMKQVCGSLKLELAQYREVAAFAQFGSDLDPATQAL        420
ORF105      YRGIRPAINVGLSVSRVGSAAQLKSMKQVCGSLKLELAQYREVAAFAQFGSDLDPATQAL        420
            ************************************************************

ORF47       LNRGARLTEVPKQPQYEPLPIEKQILVIYAAVNGFCDRMPLDRIPQYERAIPSSIKPELL        480
ORF105      LNRGARLTEVPKQPQYEPLPIEKQILVIYAAVNGFCDRMPLDRIPQYERAIPSSIKPELL        480
            ************************************************************

ORF47       KELKSGLMWISIHSFFTYGPLYYIVNWWVGQFLSFLLSKLKSGFGKWLKREQSPLAESAP        540
ORF105      KELKSGLTNERKRELDEF-----------------LLQQTKNIT----------------        507
            *******     :.:  :                 **.: *.

ORF47       IYYWLYYILLAMLLQEVPCVAACDEVGVHLLTSPPIIDAGAPVEAPEVPPAAPDIPFLEQ        600
ORF105      ------------------------------------------------------------        507

ORF47       PLLPDNEREDELYRRFLANTWGEEPTRRRIEETIRLQSEVERRIEAALVADGFDPDQVLS        660
ORF105      ------------------------------------------------------------        507

ORF47       NRHQFRAALFYPQGRALSIATYRIYLNNISRYGTRDTRSYQRLIRYIRWDLF        712
ORF105      ----------------------------------------------------        507
```

Figure S18. Protein alignment between the two open reading frames ORF47 (M3) and ORF105 (M1) of marama mitochondrial gene *atp1*. The ORF of *atp1* on circle 313 (M3) is 205 amino acids longer than the one on circle LS1a2 (M1). The query coverage is 68% with an identity 96.65%.
